# Proteotranscriptomics Reveal Signaling Networks in the Ovarian Cancer Microenvironment*[Fn FN2]

**DOI:** 10.1074/mcp.RA117.000400

**Published:** 2017-11-15

**Authors:** Thomas Worzfeld, Florian Finkernagel, Silke Reinartz, Anne Konzer, Till Adhikary, Andrea Nist, Thorsten Stiewe, Uwe Wagner, Mario Looso, Johannes Graumann, Rolf Müller

**Affiliations:** From the ‡Institute of Pharmacology, Biochemical-Pharmacological Center (BPC), Philipps University, Marburg, Germany 35043;; §Department of Pharmacology, Max-Planck-Institute for Heart and Lung Research, Bad Nauheim, Germany 61231;; ¶Institute of Molecular Biology and Tumor Research (IMT), Center for Tumor Biology and Immunology (ZTI), Philipps University, Marburg, Germany 35043;; ‖Clinic for Gynecology, Gynecological Oncology and Gynecological Endocrinology, Center for Tumor Biology and Immunology (ZTI), Philipps University, Marburg, Germany 35043;; **Biomolecular Mass Spectrometry, Max-Planck-Institute for Heart and Lung Research, Bad Nauheim, Germany 61231;; ‡‡Genomics Core Facility, Center for Tumor Biology and Immunology (ZTI), Philipps University, Marburg, Germany 35043;; §§Clinic for Gynecology, Gynecological Oncology and Gynecological Endocrinology, University Hospital of Giessen and Marburg (UKGM), Marburg, Germany 35043;; ¶¶Bioinformatics, Max-Planck-Institute for Heart and Lung Research, Bad Nauheim, Germany 61231

## Abstract

Ovarian cancer is characterized by early transcoelomic metastatic spread via the peritoneal fluid, where tumor cell spheroids (TU), tumor-associated T cells (TAT), and macrophages (TAM) create a unique microenvironment promoting cancer progression, chemoresistance, and immunosuppression. However, the underlying signaling mechanisms remain largely obscure. To chart these signaling networks, we performed comprehensive proteomic and transcriptomic analyses of TU, TAT, and TAM from ascites of ovarian cancer patients. We identify multiple intercellular signaling pathways driven by protein or lipid mediators that are associated with clinical outcome. Beyond cytokines, chemokines and growth factors, these include proteins of the extracellular matrix, immune checkpoint regulators, complement factors, and a prominent network of axon guidance molecules of the ephrin, semaphorin, and slit families. Intriguingly, both TU and TAM from patients with a predicted short survival selectively produce mediators supporting prometastatic events, including matrix remodeling, stemness, invasion, angiogenesis, and immunosuppression, whereas TAM associated with a longer survival express cytokines linked to effector T-cell chemoattraction and activation. In summary, our study uncovers previously unrecognized signaling networks in the ovarian cancer microenvironment that are of potential clinical relevance.

High grade serous ovarian adenocarcinoma (HGSOC)^1^ is the most common ovarian cancer subtype and the most lethal of all gynecologic malignancies. It ranks fifth as the cause of death from cancer in women (1). Although most HGSOCs are highly sensitive to first-line adjuvant chemotherapy, the disease has an overall 5-year survival rate of less than 40%. Several features characteristic of HGSOC contribute to its fatal nature, including the shedding of tumor cells at a very early stage of the disease, their spreading to other pelvic and peritoneal organs via the peritoneal fluid to form transcoelomic metastases, and the tumor-promoting and immune suppressive effect of the peritoneal tumor microenvironment (TME) (2).

The peritoneal fluid, occurring as malignancy-associated ascites in the majority of HGSOC patients, contains large numbers of tumor cell spheroids with tumor-initiating “stem-like” properties, tumor-associated macrophages (TAM) and tumor-associated CD8^+^ T cells (TAT) (3–5). Tumor cell spheroids are likely to play a pivotal role in transcoelomic metastasis, because they can adhere to, and invade into, the serous membranes of the peritoneum and the omentum. This involves complex interactions with the mesothelium and the underlying extracellular matrix (ECM), which are only partially understood (5–7). Furthermore, spheroids are thought to contribute to chemotherapy failure by transiently entering a chemoresistant state characterized by low proliferative and metabolic activity, thereby providing a protective niche (2).

TAM of the HGSOC microenvironment are derived from resident peritoneal macrophages and/or blood monocytes (8). They do not exert antitumor activities, but rather promote cancer progression, tumor growth and metastasis as well as immune suppression (9–12). Consistent with their tumor-promoting functions, TAM expressing high levels of the scavenger receptor CD163 and the mannose receptor CD206/MRC1 are immunosuppressive and predictive of an early relapse of ovarian carcinoma after first-line therapy (13–15). The presumably central role of TAM in cancer cell adhesion and invasion remains a largely unresolved question.

The HGSOC microenvironment harbors different types of TAT with opposing effects on tumor progression and disease outcome (16, 17). Although the accumulation of intra-tumoral CD8^+^ T cells in ovarian carcinoma patients is strongly associated with increased levels of IFNγ and a longer survival (17–19), the T-regulatory cell (Treg) subpopulation of tumor-associated CD4^+^ T cells is instrumental in immunosuppression (20, 21) and linked to an adverse clinical course (16, 22). Ascites has strong inhibitory properties on CD8^+^ T-cell activation and promotes Treg formation, in part through immunosuppressive cytokines, such as IL-10 and TGFβ (23). However, the network of immune modulatory mediators impinging on T cells is far from understood.

Elucidating the intricate signaling network among these different cell types of the HGSOC microenvironment is essential to understand their contributions to tumor growth, progression and response to therapies. Prerequisite protein expression analyses of the HGSOC microenvironment are, however, not available to date. Here, we present the results of global, cell type-specific, parallel proteomic and transcriptomic analyses of tumor cell spheroids, TAT and TAM in the peritoneal HGSOC microenvironment. Our integrated study reveals multiple, previously unrecognized intercellular signaling pathways in the HGSOC microenvironment, which control cancer cell adhesion, invasion and metastasis as well as immunosurveillance, and are strongly associated with patient survival.

## EXPERIMENTAL PROCEDURES

### 

#### 

##### Patient Samples

Ascites was collected from patients with histologically verified HGSOC undergoing primary surgery at the University Hospital in Marburg. Informed consent was obtained from all patients according to the protocols approved by the local ethical committee. For transcriptomic analyses, cells from 33 patients were analyzed; for proteomic analyses, cells from 9 patients were analyzed. Patient characteristics are presented in supplemental Table S1. Clinical courses were evaluated by RECIST criteria (24) and profiles of serum CA125 levels (25), according to the recommendations by the Gynecologic Cancer InterGroup (GCIG).

##### Isolation of Cells from Ovarian Cancer Ascites

TAM and TAT were isolated by density gradient centrifugation followed by magnetic cell sorting (MACS) of CD14^+^ cells as described (8). Tumor spheroids were separated by filtration as previously reported (8) resulting in spheroids designated giant (>100 μm = “G”), large (>40 μm and <100 μm = “L”), medium (>30 μm and <40 μm = “m”) or small (<30 μm = “s”; including single tumor cells). The latter were further enriched by MACS depletion of CD45^+^ immune cells. All isolated cells were immediately analyzed by flow cytometry for purity or lysed for RNA preparation as described (8).

##### Flow Cytometry Analysis of Receptors and Intracellular Growth Factors and Cytokines in TAM

FITC-labeled anti-TGFβ-RIII (R&D Systems, Minneapolis, MN; cat.# FAB242F), PE-labeled anti-LIF-R (R&D Systems; Clone 32953; cat.# FAB249P) and PE-labeled anti-IL-10R (Milteny, Bergisch Gladbach, Germany; clone REA239; cat.# 130–101-542) were used for surface staining. Intracellular staining of permeabilized cells was performed as described previously (15) with APC-labeled anti-IL-8 (eBioscience, Frankfurt am Main, Germany; Clone 8CH; cat.# 17–8088-41/42), FITC-labeled anti-S100A8/A9 (Life Technologies, Carlsbad, CA; Clone CF-145; cat.# MA5–17623), PE-labeled IL-1β (eBioscience; Clone CRM56; cat.# 12–7018), PE-labeled IL-10 (BD Pharmingen, San Jose, CA; Clone JES3–19F1; cat.# 554706) and PE-labeled TGFβ1 (BD Pharmingen; Clone TW4–9E7; cat.# 562339). Isotype control antibodies were purchased from BD Biosciences, Miltenyi Biotech and eBioscience. Cells were analyzed by flow cytometry and results were calculated as percentage of positive cells and mean fluorescence intensities (MFI). Flow cytometry was carried out on a FACS Canto II and data were analyzed using Diva (BD Biosciences).

##### Databases and Data Resources

All genome sequence, gene and protein annotation was retrieved from Ensembl 81 (26). Functional annotations were performed by PANTHER gene ontology (GO) enrichment analysis (27) (http://www.geneontology.org). In case of redundancies in the search results (*e.g.* “regulation of chemotaxis” (GO:0050920), “positive regulation of chemotaxis” (GO:0050921), “regulation of leukocyte chemotaxis” (GO:0002688), “cell chemotaxis” (GO:0060326) only the term with the highest enrichment and significance was included in the Figs. 2*B*, 3*B*, 4*A*, 4*B*, and 7*A*. Overall survival (OS) data were retrieved from PRECOG (*PRE*diction of *C*linical *O*utcomes from *G*enomic Profiles; https://precog.stanford.edu) (28), a database for querying associations between genomic profiles and cancer outcomes.

**Fig. 1.**
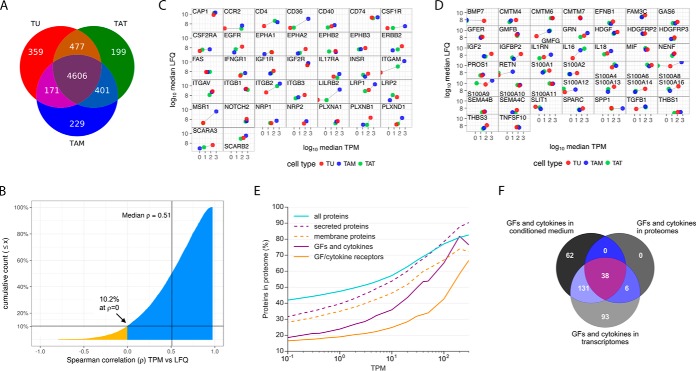
**Analysis of the proteome and transcriptome of TU, TAM and TAT.** *A*, Venn diagram showing the number of proteins expressed or coexpressed by TU, TAM and TAT with a median LFQ intensity greater that the 0.25 quantile of the combined proteome (LFQ intensity>50 × 10^6^). *B*, Data of all patients and all cell types were analyzed to calculate the cumulative distribution of Spearman correlation coefficients (ρ) between mRNA (TPM) and protein (LFQ intensity) expression values, resulting in a median value of ρ = 0.51. The blue area indicates positive correlations (89.8% of all instances), yellow indicates negative correlations (10.2%). *C*, *D*, Correlation of protein and mRNA expression in TU (red), TAM (blue), and TAT (green) for all growth factors/cytokine receptors (*C*) or growth factors/cytokines (*D*) present in the proteome and transcriptome of at least one cell type. LFQ intensity values were plotted against TPM. Connecting lines with a positive slope indicate positive correlation of mRNA and protein expression, connecting lines with a negative slope indicate negative correlation. See supplemental Figs. S5 and S6 for further analyses. *E*, Percentage of genes for which protein expression was detected (*n* = 7186) plotted against mRNA expression levels (TPM) of the genes, using data from all patients and all cell types. *p* values: receptors or growth factors/cytokines *versus* random sets of proteins (see simulations in Supplemental Fig. S4 for details). *F*, Venn diagram showing the number of growth factors and cytokines detected in the transcriptomes (TPM>0.3), the proteomes (LFQ intensity>50 × 10^6^), or conditioned media (found in ≥50% of the samples) of TU and TAM.

Subcellular location information was downloaded from the Human Protein Atlas (http://www.proteinatlas.org/download/subcellular_location.csv.zip). Predicted intracellular proteins were identified by protein class “Predicted intracellular protein.” Proteins predicted as predominantly or exclusively membrane-associated (used in Fig. 1*E* and supplemental Fig. S2*A*) were identified by the presence of the term “membrane” and the absence of the term “intracellular” in protein class (*n* = 3514). A list of predicted secreted proteins (*n* = 2253; used in Fig. 1*E* and supplemental Fig. S2*A*) was compiled by calculating the intersections of four data sets: “Predicted secreted proteins,” “SignalP predicted secreted proteins,” “Phobius predicted secreted proteins,” “SPOCTOPUS predicted secreted proteins” downloaded from the Human Protein Atlas:

http://www.proteinatlas.org/search/protein_class:Predicted%20secreted%20proteins

http://www.proteinatlas.org/search/protein_class:SignalP%20predicted%20secreted%20proteins

http://www.proteinatlas.org/search/protein_class:Phobius%20predicted%20secreted%20proteins

http://www.proteinatlas.org/search/protein_class:SPOCTOPUS%20predicted%20secreted%20proteins

**Fig. 2.**
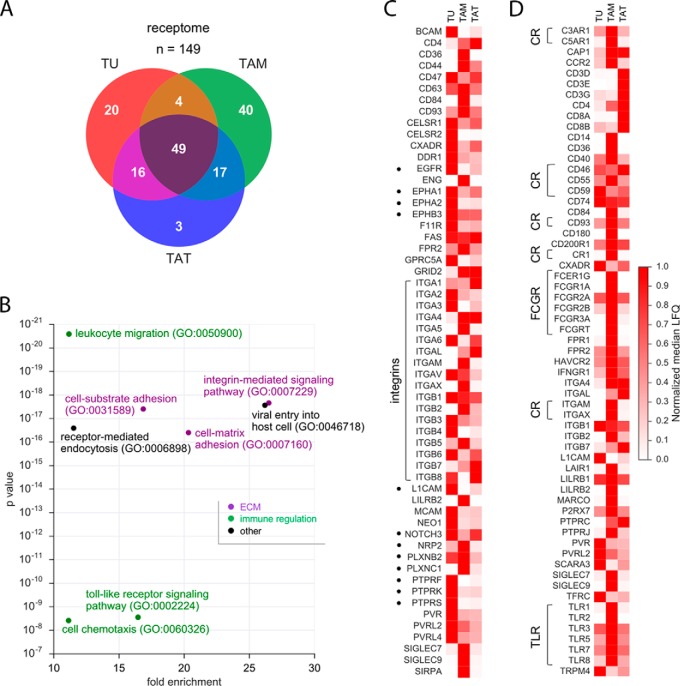
**The receptome of TU, TAM and TAT.** *A*, Venn diagram showing the number of membrane receptors present in the proteomes of TU, TAM and/or TAT (LFQ intensity> 50 million). *B*, PANTHER functional annotation (GO enrichment analysis) of membrane receptors expressed in TU, TAM or TAT. The figure shows the top non-redundant terms by enrichment (>10-fold) and *p* value (<10^−7^). Different functional classes are represented in different colors. (c, d) Heatmaps showing the expression of membrane receptors listed in the GO term set “cell adhesion” (*c*) or “immune response” (*d*) in TU, TAM and TAT. For each receptor, the median LFQ intensity value of the cell type with the highest expression was set to 1; the median LFQ intensity values of the other cell types were normalized accordingly. CR: complement receptors; TLR: toll-like receptors; FCGR: receptor for the Fc region of immunoglobulins gamma; dots: receptors with known functions in cancer biology.

A data set of all human plasma membrane receptors (supplementary Data set S5; used for Figs. 1*E* and 2, supplemental Fig. S2*A*) was built as follows: First, we identified all genes in the HUGO database with the term “receptor” in their description. From this set, false positives were removed by excluding all genes with the following terms in their description: “putative,” “accessory,” “assoc,” “bound,” “chemosensory,” “cornichon,” “endoplasmic reticulum,” “golgi,” “interact,” “intracellular,” “non-receptor,” “nuclear,” “orphan,” “peroxisome,” “regulat,” “retinoic,” “retinoid,” “signal recognition particle,” “signal sequence,” “steroid,” or “substrate.” The resulting list (supplementary Data set S6) was combined with a receptor list published by Ramilowski *et al.* (29) manually curated using GeneCards database information (supplementary Data set S7)and a list of CD markers representing membrane receptors (supplementary Data set S8).

Lists of all growth factors/cytokines, their cognate receptors, enzymes synthesizing lipid mediators and lipid receptors were retrieved from (30). The sets of growth factors/cytokines and their receptors were updated using the GeneCards database (http://www.genecards.org). This data was used to define groups of growth factor/cytokine receptors and their interacting ligands (supplementary Data set S10*A*; used for Figs. 1*C*, 1*D*, 1*E*, 1*F*, 5, 6, 7, supplemental Figs. S2*B*, S3, S4, S5, S6).

##### RNA Sequencing (RNA-Seq) and Analysis of RNA-Seq Data

RNA isolation and RNA-Seq was carried out on an Illumina HiSeq 1500 as described using the Illumina TruSeq stranded total RNA kit (30). RNA-Seq data were aligned using STAR (version 2.3.1z13_r470) and processed as reported (30). TPM (transcripts per million) were calculated based on the total gene read counts, and corrected for contamination by tumor cells as described (30). Genes were considered expressed if they had a minimum TPM of 2 (except for Fig. 1*F*: TPM>0.3). Coexpression analyses (Figs. 6 and 7) were carried out on genes showing a high variance of expression (expression >median variance) as determined by the Python function *pandas.DataFrame.var ( )*.

##### Protein Mass Spectrometry

Isolated and pelleted tumor spheroids, TAM and TAT were lysed in SDS buffer (4% SDS in 0.1 m Tris/HCl pH 7.6) before heating samples at 95 °C for 5 min. DNA was sheared by sonication and cell debris was removed by centrifugation at 16.000 g for 10 min. DC protein assay (BioRad, Hercules, CA) was used to determine concentration of solubilized proteins in supernatants after centrifugation. Equal amounts of proteins (40 μg each) were loaded on a gradient gel (NuPAGE 4–12% Bis-Tris gel, Invitrogen, Carlsbad, CA) and separated by SDS-PAGE before in-gel digestion (32).

For proteomic analyses of conditioned media, tumor spheroids and TAM freshly prepared from ascites of 5 HGSOC patients were cultured in autologous cell-free ascites for 16 h at 37 °C and 5% CO_2_. After that, the ascites was aspirated, and the cells were washed three times in PBS and twice in serum-free medium. Cells were cultured in serum-free medium for another 5 h before harvesting the culture supernatants for proteomic analysis as described below. Tumor spheroids were cultured in serum-free M199 (Gibco, Carlsbad, CA) mixed 1:2 with DMEM/Ham's F-12 (1:1; Biochrom, Berlin, Germany). Serum-free RPMI1640 supplemented with 2 mm l-alanyl-l-glutamine (Gibco) was used for culture of TAM. Up to 40 μg of proteins were loaded on a gradient gel (NuPAGE 4–12% Bis-Tris gel, Invitrogen) and separated by SDS-PAGE before in-gel digestion (32).

Following separation by SDS-PAGE, gel lanes were cut in 10 pieces and proteins were reduced (10 mm dithiothreitol), alkylated (55 mm iodoacetamide) and digested by trypsin (Promega, Mannheim, Germany), using an enzyme to protein ratio of 1:100. After overnight incubation, peptides were gradually eluted by increasing amount of acetonitrile before sample desalting by stop and go extraction (STAGE) tips (33).

For mass spectrometry (MS) analysis, peptides were eluted from STAGE tips by solvent B (80% acetonitrile, 0.1% formic acid), dried down in a SpeedVac Concentrator (Thermo Fisher Scientific, Waltham, MA) and dissolved in solvent A (0.1% formic acid). Peptides were separated using an UHPLC (EASY-nLC 1000, ThermoFisher Scientific) and 20 cm, in-house packed C18 silica columns (1.9 μm C18 beads, Dr. Maisch GmbH, Ammerbuch, Germany) coupled in line to a QExactive HF orbitrap mass spectrometer (ThermoFisher Scientific) using an electrospray ionization source. A gradient was applied using a linearly increasing concentration of solvent B (80% acetonitrile, 0.1% formic acid) over solvent A (0.1% formic acid) from 10% to 38% for 55 min and from 38% to 60% for 5 min, followed by washing with 95% of solvent B for 5 min and re-equilibration with 5% of solvent B.

Full MS spectra were acquired in a mass range of 300 to 1750 *m*/*z* with a resolution of 60,000 at 200 *m*/*z.* The ion injection target was set to 3 × 10^6^ and the maximum injection time limited to 20 ms. Ions were fragmented by high-energy collision dissociation (HCD) using a normalized collision energy of 27 and a ion injection target of 1.0 × 10^5^ with a maximum injection time of 25 ms. The resulting tandem mass spectra (MS/MS) were acquired with a resolution of 15,000 at 200 *m*/*z* using data dependent mode with a loop count of 15 (top15).

##### Experimental Design and Statistical Rationale of Proteomic Analyses

Raw MS data from 27 *ex vivo* samples (matching TU, TAM, and TAT samples from 9 patients), and from conditioned media of 5 *ex vivo* samples (matching TU and TAM) was analyzed using MaxQuant 1.5.5.1 (Cox & Mann, 2008) in label free quantitation mode (LFQ), which includes the Andromeda search engine. The database used was Ensembl 81 containing 101,933 sequences. Parameters differing from default values were as follows: minimum of 1 unique peptide per protein group (UniquePeptides = 1), usage of “match between runs” (matchBetweenRuns = true) with a matching time window of 0.7 min (matchingTimeWindow = 0.7) and an alignment time window of 20 min (alignmentTimeWidow = 20); ibaq = true, ibaqLogFit = true. The protease used was trypsin, the only fixed modification included was carbamidomethyl, variable modifications permitted were oxidation and N-terminal acetylation (“acetyl”). There were 2 missed or nonspecific cleavages permitted, the mass tolerance for precursor ions was 4.5 ppm and the mass tolerance for fragment ions was set to 20 ppm. Protein groups identified by MaxQuant were matched to their respective genes. Groups matching multiple genes were duplicated to match protein and RNA data of genes that are annotated multiple times in Ensembl (such as LILRA4). This led to the amplification of a small number of gene families (histones, PRAME family members, Peptidylprolyl Isomerase A Like family members, and keratin associated proteins), which were therefore not considered in gene enrichment analyses. Missing LFQ intensity values (“0′ in MaxQuant output) were replaced by a minimum non-zero value with a randomized Gaussian relative error of 0.59 added (0.59 was the overall relative standard deviation across all protein groups).

False discovery rate estimation was performed according to the MaxQuant implementation of the concatenated reverse database approach proposed by Peng *et al.* (34, 35). FDR requirements were 1% on both the peptide and protein group identification levels.

**Fig. 3.**
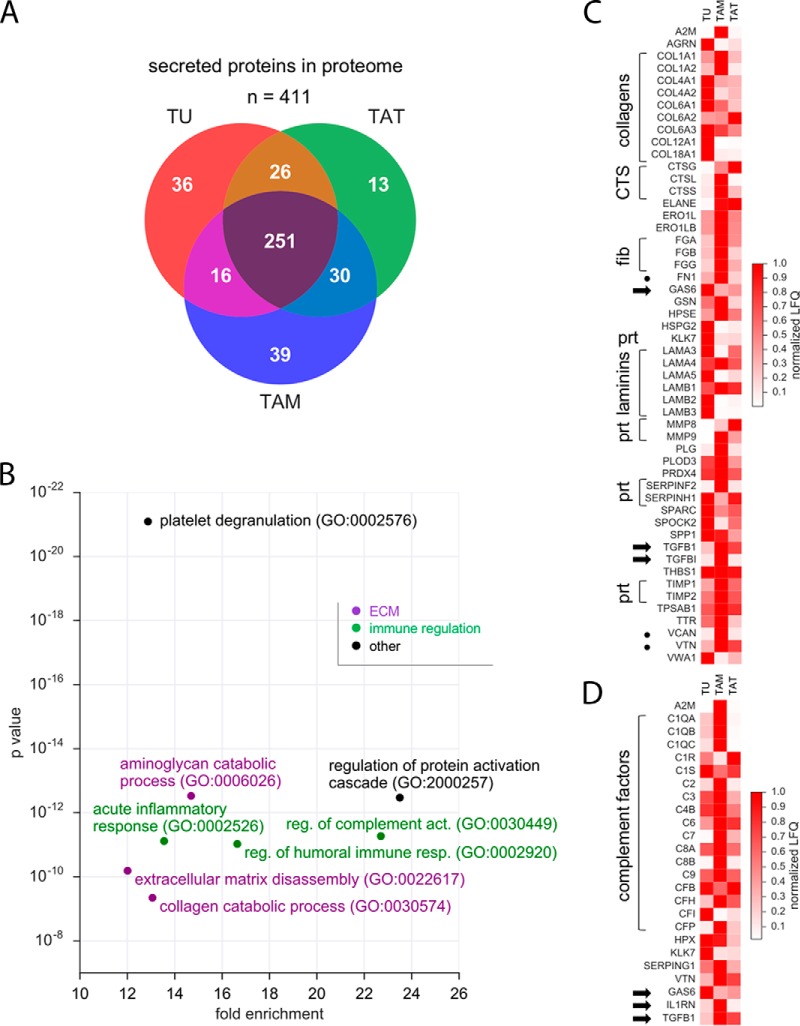
**The secretome of TU, TAM and TAT.** *A*, Venn diagram showing the number of predicted secreted proteins in the proteomes of TU, TAM and/or TAT. *B*, PANTHER functional annotation of all secreted proteins identified in the proteomes of TU, TAM or TAT. The figure shows the top non-redundant terms by enrichment (>10-fold) and *p* value (<10^−7^). *C*, *D*, Heatmaps showing the expression of secreted proteins listed in the GO term set “ECM organization” (*C*) or “immune response” (*D*). For each secreted protein, the median LFQ intensity value of the cell type with the highest expression was set to 1; the median LFQ intensity values of the other cell types were normalized accordingly. CTS: cathepsins; fib: fibrinogens; prt: proteases and protease inhibitors. Dots: ECM proteins; arrows: growth factors/cytokines.

Purity of protein samples assessed by cell type-specific markers (supplemental Fig. S1*A*) revealed a high contamination of one TAT and one TAM sample with TU proteins (not shown), *i.e.* tumor marker expression at tumor cell levels. These samples (TAT92 and TAM108) were excluded from the present study. Three TAT samples with lower tumor cell contamination (TAT08, TAT114, TAT133; triangles in supplemental Fig. S1*A*; supplemental Table S1) were excluded only from the analysis of TU-specific expression in Fig. 3 and supplemental Fig. S1*C*. Each of these three TAT samples had a higher sum of LFQ intensities of tumor markers (sum of LFQ intensities of EPCAM, PAX8, MSLN, MUC16, and ITGB4) than the TU sample with the lowest sum of LFQ intensities of tumor markers.

Principal component analysis (supplemental Fig. S1*B*) was performed using the Python module sklearn on rescaled LFQ data (using the MinMaxScaler). For the Venn diagram in Fig. 1*A*, proteins were considered expressed if their median LFQ intensity reached the 0.25 quantile of the combined proteome (= 50 million). supplemental Fig. S1*C* is based on all samples shown in supplemental Fig. S1*A*; for identifying TU-specific proteins in the TAT *versus* TU comparison, samples labeled “contaminated” were excluded. In supplemental Fig. S1*C*, statistically significant fold changes were tested using an unpaired permutation approach (*i.e.* not assuming a specific distribution) using the Python permute framework for 100,000 iterations, and genes were marked red if they had significant (FDR <0.05) and at least 2-fold difference.

For Fig. 1*C*, proteins were considered “in proteome” if there was a protein group (identified in any sample by MaxQuant) associated to them. *p* values were calculated by drawing random subsets of “all” with the same size as the query set (without replacement). A “random subset curve” was considered equal or better than the query curve if it had more missing proteins at any TPM threshold at which the query curve had at least 20 genes remaining. The *p* value was defined as the number of random curves drawn that were equal or better than the query curve divided by the number of random curves drawn. The simulations are visualized in supplemental Fig. S4.

To minimize false positives in the analyses in Figs. 2*A* and 3*A* arising from potential cross-contaminations among cell types, proteins were considered expressed in a given cell type if their median LFQ intensity value was >50 million and maximally 5-fold higher in any of the other two cell types.

As surrogate markers for survival (supplemental Fig. S8), we used all genes with a PRECOG z-score of >4.0 or <-4.0 (28). Coexpression analysis was carried out for proteins encoded by these surrogate marker genes achieving a minimum Spearman correlation with RNA expression of ρ>0.3. The expression of these surrogate marker proteins across all 9 TU samples was correlated with the entire proteome after standardization of LFQ intensities for each marker to range of 0–1.0. Surrogate markers yielding Spearman correlation coefficients ρ>0.95 were grouped. Only groups with at least 8 proteins were considered further (supplemental Fig. S8*A*).

##### Other Statistical Analyses

Spearman correlation coefficients (ρ) and *p* values were determined by *scipy.stat.spearmanr (Python*). Significance levels are as ****, ***, ** and * for *p* < 0.0001, *p* < 0.01, *p* < 0.01 and *p* < 0.05, respectively.

## RESULTS

### 

#### 

##### Proteotranscriptomic Analysis of the HGSOC Microenvironment

In a first step toward understanding the signaling networks in the HGSOC microenvironment, we determined the proteome of tumor cell spheroids (TU, *n* = 9), TAM (*n* = 8) and TAT (*n* = 8) isolated from the ascites of HGSOC patients (supplementary Note, supplemental Fig. S1, supplementary Data sets S1 and S2). In total, we identified 7186 proteins expressed by TU, TAM, and TAT, of which 6442 proteins showed a median label-free quantification (LFQ) intensity >50 million (corresponding to the 0.25 quantile of the combined proteome). 4606 of these were found expressed in each cell type, whereas 359, 229, and 199 proteins were selective for TU, TAM, or TAT samples, respectively (Fig. 1*A*). In our effort to obtain a comprehensive picture of the functionally relevant signaling networks in the HGSOC microenvironment, we next determined the transcriptome of TAT (*n* = 6) and increased the sample sizes of a previous study of TU and TAM (30) to *n* = 23 and *n* = 29, respectively. This yielded a total of 19 samples (TU = 8, TAM = 8, TAT = 3), for which both transcriptomic as well as proteomic data were available (supplemental Table S1). Consistent with the generally higher detection limit of proteomic compared with transcriptomic technologies (*e.g.* ref (36).), we identified a lower number of proteins by mass spectrometry (*n* = 7,186) than mRNAs by RNA-Seq (*n* = 14,128, transcripts per million (TPM)>2; supplementary Data set 3). This suggests that the construction of molecular signaling networks based on proteomic data may lack essential components, and that these lacking components could be complemented by transcriptomic data. To address this possibility, we first analyzed the correlation between mRNA and protein abundance of TU, TAM and TAT. The cumulative distribution of Spearman correlation coefficients between mRNA (TPM) and protein (LFQ intensity) expression values was calculated for all samples and cell types, showing positive correlations for 89.8% of all instances, negative correlations for 10.2% and a median Spearman′s ρ value of 0.51 (Fig. 1*B*; genes with a particularly high correlation are listed in supplementary Data set S4), clearly indicating a good correlation of mRNA and protein abundance.

Next, we examined the overlap between transcriptome and proteome for sets of proteins with different subcellular localizations. Toward this goal, we first compiled data sets for all predicted membrane receptors (supplementary Data set S5; based on supplementary Data set S6-S8 as described under Experimental Procedures), as well as for all predicted secreted proteins (supplementary Data set S9). Because a major focus of our study was the investigation of intercellular signaling pathways in the HGSOC microenvironment, we additionally generated a database of 136 groups of growth factor and cytokine receptors (*n* = 257) and their ligands (*n* = 466), as well as a set of ligands for which receptors are not known (orphan ligands; *n* = 47), using published data and publicly accessible databases (supplementary Data set S10*A*; see Experimental Procedures for details). The term “growth factors and cytokines,” as used in this manuscript, also includes polypeptides with growth factor-like signaling functions, such as hormones, neuroregulators and axon guidance molecules. To study the correlation between transcriptome and proteome for genes encoding growth factor/cytokine receptors and their ligands in a cell type-specific manner, we plotted LFQ intensity values against TPM for each of the protein/mRNA pairs. Indeed, we observed a positive correlation of mRNA and protein expression levels in most cases (Figs. 1*C* and 1*D*). For example, the rank order for EGFR or ERBB2 expression is TU > TAT > TAM (Fig. 1*C*), or for IL16 TAT > TAM > TU (Fig. 1*D*) for both mRNA and protein. These data corroborate the good correlation between mRNA and protein abundance for the different cell types of the HGSOC microenvironment.

As expected, the proportion of detected proteins increased with increasing mRNA levels (Fig. 1*E*, analysis across all three cell types, TU, TAM, and TAT). However, protein detection probabilities were clearly reduced for genes encoding membrane or secreted proteins (Fig. 1*E*). This discrepancy decreased at higher mRNA expression levels (Fig. 1*E*). Compared with membrane or secreted proteins, protein detection probabilities for growth factor/cytokine receptors or their ligands were even further reduced (Fig. 1*E*; *p* ≤ 0.022 and *p* ≤ 0.0006, respectively; significance tests in supplemental Fig. S4). For example, for genes with an mRNA expression value of TPM = 1, overall ∼50% of the corresponding proteins could be detected; however, if those genes encoded growth factors or cytokines, only 25% of the corresponding proteins were found in the proteome.

We next performed proteomic analyses of conditioned media of TU and TAM after a 5-hour culture in protein-free medium. In these culture supernatants, we identified 1528 secreted proteins (detected in at least 50% of the samples; supplementary Data set S11; secreted proteins as defined in supplementary Data set S9). As shown in Fig. 1*F*, 86.4% (*n* = 38) of the 44 growth factors/cytokines in the proteomes of TU and TAM could also be detected in the conditioned media of TU and TAM. Furthermore, out of 224 growth factors/cytokines detected in the transcriptomes, but not in the proteomes of TU and TAM, 131 (58.5%) were found in the conditioned media of TU and TAM. The conditioned media also contained 62 growth factors/cytokines not present in the transcriptomes, which we attribute to a highly efficient translation and secretion of weakly expressed genes (below the applied cut-off of TPM>0.3) as well as to the up-regulation of a subset of genes under the culture conditions.

Our results suggest that there are at least three reasons contributing to the underrepresentation of growth factors/cytokines and their receptors in the proteomes. These are (1) relatively low expression levels of secreted and membrane proteins, and in particular low intracellular expression levels of secreted proteins (2) a generally lower detectability of secreted and membrane proteins (*e.g.* because of a lower frequency of arginines and lysines in transmembrane domains, and/or because of a higher hydrophobicity of transmembrane domain peptides that interferes with LC separation), as previously reported (37–40) and (3) heterogeneity among cells with only a small percentage of cells expressing a particular secreted or membrane protein at a detectable level. Taken together, our results indicate that transcriptomic data are suitable to complement proteomic data, for secreted growth factors and cytokines.

##### The Receptomes and Secretomes of the HGSOC Microenvironment Point to Cell Type-selective Functions in Integrin-mediated Adhesion and Immune Regulation

To elucidate the intercellular communication networks in the HGSOC microenvironment, we next analyzed the proteome-derived receptomes and secretomes, *i.e.* all predicted membrane receptors and secreted proteins (as defined in supplementary Data set S5 an S9). We identified 149 membrane receptor proteins expressed by at least one cell type (supplementary Data set S15; LFQ intensity >50 million). Of these, 20 were selective for TU, 40 for TAM and 3 for TAT; 49 membrane receptor proteins were found in all three cell types (Fig. 2*A*). GO enrichment analysis of membrane receptors expressed in TU, TAM or TAT revealed pathways involved in remodeling of the extracellular matrix (ECM) and cell adhesion as well as pathways regulating the immune response (Fig. 2*B*).

The most prominent proteins of the “cell adhesion” group were integrins (ITG; Fig. 2*C*) and receptors with pivotal signaling functions in cancer biology, including ephrin receptors (EPH), EGFR, NOTCH3, the adhesion molecule L1CAM, receptor-type tyrosine-protein phosphatases (PTPR) as well as semaphorin receptors of the neuropilin (NRP) and plexin (PLXN) families (marked by dots in Fig. 2*C*). Numerous of these receptor proteins are expressed in a cell type-selective manner by TU and TAM, pointing to a cooperation of these cell types in adhesion and invasion. The major constituents of the “immune response” group were complement receptors (CR), immunoglobulin receptors (FCGR) and toll-like receptors (TLR), mostly expressed at highest levels by TAM (Fig. 2*D*).

To identify ligands, which could activate these receptors in the HGSOC environment, we performed a similar analysis of the secretome, *i.e.* for all predicted secreted proteins, and identified 411 proteins in the proteomes of TU, TAM and TAT (Fig. 3*A*; LFQ intensity >50 million; supplementary Data set S16). Functional annotation revealed GO terms linked to platelet degranulation, ECM organization and immune regulation, the latter including “regulation of complement activation” as top hits (Fig. 3*B*). The most conspicuous proteins of the “ECM organization” group were ECM constituents, including fibrinogens (FG), fibronectin (FN), laminins (LMN), versican (VCAN) and vitronectin (VTN), proteases of the cathepsin (CTS), kallikrein (KLK) and matrix metalloprotease (MMP) families as well as proteinase inhibitors of the SERPIN and TIMP subgroups (Fig. 3*C*), whereas the “immune regulation” group contained almost all components of the complement system (Fig. 3*D*).

Because many growth factors and cytokines expressed at the transcriptional level were not detectable in the proteomes (Fig. 1*D*), we analyzed proteomic data of conditioned media from TU and TAM (see above). Table I lists the most abundant growth factors and cytokines in these culture supernatants. These include many ligands regulating major signaling pathways, such as IGF, JAG (NOTCH ligands), S100, SEMA, SLIT, THBS (thrombospondin), VEGF, and WNT.

**Table I:** Proteins secreted by TU or TAM cultured for 5 h in protein-free me-dium. The table lists the most abundant proteins (LFQ intensity > median LFQ intensity of all proteins, and present in at least 50% of the samples). Note that TU and TAM values are not directly comparable because of the lack of normalizers

Name	TU median (LFQ/10^7^)	TAM median (LFQ/10^7^)	Name	TU median (LFQ/10^7^)	TAM median (LFQ/10^7^)
**ANGPT1**	18.7	22.4	**NRG2**	21.6	127.9
**BMP1**	118.3	159.0	**NTF4**	11.9	9.2
**BMP3**	14.2	26.2	**OGN**	43.1	26.4
**CCL18**	8.4	86.6	**PROS1**	126.8	357.3
**CMTM1**	35.2	64.9	**S100A2**	16.1	12.4
**CTGF**	23.4	13.3	**S100A4**	59.9	32.7
**CX3CL1**	16.6	17.7	**S100A6**	245.5	97.9
**CXCL5**	12.1	88.1	**S100A8**	14.2	1353.4
**CXCL8**	12.0	23.6	**S100A9**	10.8	285.9
**CXCL16**	0.0	28.6	**S100A10**	25.5	15.1
**DLL1**	28.9	25.9	**S100A11**	80.4	149.4
**DLL4**	13.2	14.5	**S100A13**	39.4	0.0
**EFEMP1**	65.9	90.4	**S100A16**	41.2	4.8
**EFNA1**	13.5	6.0	**SEMA3C**	8.5	22.4
**EGF**	28.1	22.9	**SEMA3D**	13.0	13.7
**FAM3C**	46.8	30.5	**SEMA3E**	25.4	21.3
**FAM65C**	27.4	18.1	**SEMA3G**	15.1	25.1
**FGF2**	4.2	14.3	**SEMA4B**	18.8	34.5
**FGF7**	30.9	23.7	**SEMA4C**	14.4	16.6
**FGF12**	11.2	14.8	**SEMA4D**	22.1	24.6
**FST**	16.2	10.3	**SEMA4F**	32.6	49.1
**FSTL1**	17.5	15.2	**SEMA5A**	17.0	17.7
**GAS6**	8.4	17.5	**SEMA5B**	7.7	14.6
**GDF1**	53.2	85.2	**SEMA6A**	11.7	10.1
**GDF9**	11.2	18.1	**SEMA6B**	8.1	13.9
**GDF15**	11.6	15.4	**SEMA6C**	28.1	35.7
**GMFB**	31.3	34.3	**SEMA7A**	396.1	341.5
**GMFG**	0.0	15.6	**SLIT1**	354.9	611.6
**GRN**	10.8	18.7	**SLIT2**	20.6	20.7
**HDGF**	101.4	47.0	**SLIT3**	28.4	43.2
**HGF**	22.0	46.0	**SPARC**	21.7	59.2
**IGF1**	63.8	56.4	**STC2**	15.6	2.5
**IGF2**	19.7	11.9	**TGFB2**	18.3	15.4
**IGFALS**	27.4	35.3	**TGFB3**	11.0	14.1
**IGFBP2**	143.7	35.2	**THBS1**	18.4	784.6
**IGFBP3**	179.4	59.4	**THBS2**	20.1	34.0
**IGFBP4**	7.3	16.7	**THBS3**	20.0	14.5
**IGFBP5**	12.5	32.4	**THBS4**	46.8	116.2
**IGFBP6**	25.5	24.6	**TNFRSF11B**	36.5	18.7
**IGFBP7**	27.1	27.6	**TNFSF10**	6.4	20.9
**IL1RN**	20.4	63.4	**TRH**	55.1	63.0
**IL16**	24.2	26.8	**VEGFA**	16.0	8.2
**IL17D**	11.8	35.5	**VEGFC**	21.6	40.1
**IL18**	11.3	11.2	**WNT2B**	10.4	20.0
**INHBA**	6.6	15.0	**WNT5A**	31.1	35.3
**JAG1**	23.6	31.8	**WNT6**	24.5	27.4
**JAG2**	46.5	97.0	**WNT7A**	13.1	12.3
**MDK**	15.7	5.2	**WNT7B**	15.3	31.2
**MIF**	62.4	30.9	**WNT10A**	14.6	19.1
**NRG1**	12.7	28.9	**WNT11**	16.0	12.4

##### Signaling Networks in the HGSOC Microenvironment Based on Cell Type-specific Transcriptomes

**Fig. 4.**
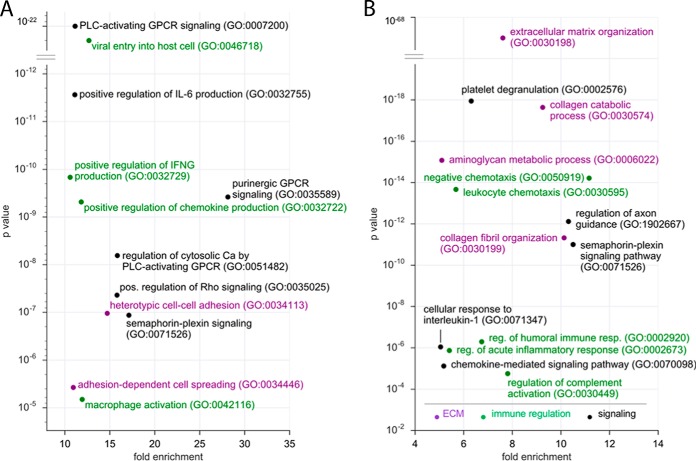
**Transcriptomics reveal signaling networks in the HGSOC microenvironment.** *A*, PANTHER functional annotation (GO enrichment analysis) of genes for predicted membrane receptor proteins and expressed in TU, TAM or TAT (*n* = 625). The figure shows the top terms by enrichment (>10-fold) and *p* value (<10^−5^). GPCR: G-protein-coupled receptor; IFNG; interferon-γ; PLC: phospholipase C. *B*, Functional annotation of genes for predicted secreted proteins and expressed in either cell type (*n* = 1168). The figure shows the top terms by enrichment (>3-fold) and *p* value (<10^−4^). Different functional classes are represented in different colors in both panels.

As our correlation analysis between transcriptome and proteome had shown that expression of membrane receptor and secreted proteins is prone to evade detection by proteomics (see above), we aimed at complementing our proteome-based analysis of signaling networks in the HGSOC microenvironment by our transcriptomic data. We therefore studied the mRNA expression of genes encoding membrane receptors or secreted proteins (as defined in (supplementary Data set S5 and S9), respectively). Functional annotation of those genes expressed in TU, TAM, or TAT showed most significant enrichments for pathways involved in G protein-coupled receptor (GPCR) signaling, axon guidance signaling, organization of the extracellular matrix and cell adhesion, immune regulation and complement activation (Figs. 4*A* and 4*B*).

**Fig. 5.**
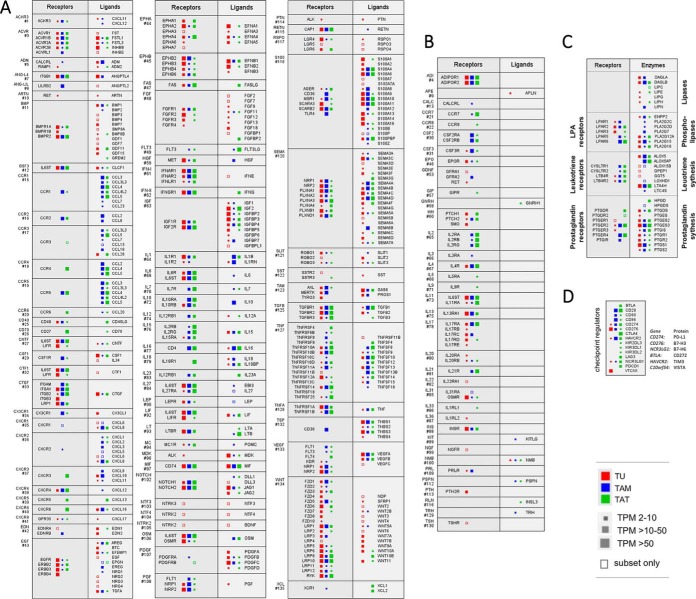
**Cellular origins and targets of signaling in the HGSOC microenvironment.** *A*, Schematic representation of growth factor- and cytokine-driven signaling pathways operating among TU (red), TAM (blue) and TAT (green). Receptors (left) and their respective ligands (right) are arranged in adjacent blocks. Numbers indicate the group ID as defined in Supplemental Data set S10A. The sizes of the filled squares in A-C indicate the level of expression determined by RNA-Seq (high: median TPM >50; intermediate: median TPM >10; low: median TPM >2). Open squares indicate cases, where substantial expression (TPM >3) was observed only in a small fraction of samples (<10%). Only pathways for which expression of both ligands as well as their cognate receptors was detected are shown. *B*, Pathways for which either only ligand or only receptor expression (but not both) could be detected. *C*, Schematic representation of lipid-mediated signaling pathways. Left: receptors; right: enzymes involved in lipid metabolism and the generation of signaling mediators, LPA and eicosanoids. *D*, mRNA expression of immune checkpoint regulators in TU, TAM and TAT.

Next, we examined the cell type-specific mRNA expression of genes for growth factor/cytokine receptors and their ligands (supplementary Data set S10*A*, S12, S13). This allowed for the construction of interaction maps showing the sources of ligands and their potentially targeted cell types (Fig. 5*A*). These data also point to a number of cell type-selective signaling components, as defined by the selective expression of a ligand or receptor by one particular cell type (Table II).

**Table II:** Cellular sources and targets of growth factors and cytokines

Cell type	Main target of	Group IDs (Fig. 5*A*)
TU	EGF via EGFR, ERBB2–4	42
	FGF via FGFR1–4	48
	HGF via MET	59
	RSPO via LGR4–6	117
	WNT via LRP	134
TAM	ANGPTL2 via LILRB2	8
	CSF1 via CSF1R	29
	S100 via CD36, MSR1, TLR4	118
	chemokines via CCR1, CCR2	15, 16
TAT	CD70 (TNFSF7) via CD27 (TNFRSF7)	26
	IL18 via IL18R1	79
	chemokines via CCR4/6, CXCR3/5/6	18, 20, 37, 39, 40
All	IFNE via IFNAR1/2	61
	IFNG via IFNGR1	62
	IGF via IGF2R	63
	IL6 via IL6R	69
	MIF via CD74	97
	DLL, JAG via NOTCH	102
	RETN via CAP1	115
	SEMA via NRP, PLXN	120
	TGFB via TGFBR	125
	TNFSF via TNFRSF	127
	WNT via LRP	134

Cell type	Main producer of	Group IDs (Fig. 5*A*)
TU	CSF1	29
	EDN1/2	43
	ephrins (EFNA/B)	44, 45
	FGF	48
	MDK	96
	S100s	118
	WNTs	134
TAM	HGF	59
	IL1B	64
	IL6	69
	IL10	72
	S100A8	118
	SEMA	120
	chemokines (CCL, CXCL), IL16, oncostatin M (OSM), TGFB1	15–20, 35–41, 77, 106, 125
TAT	CD40LG	25
	FASLG	47
	FLT3LG	49
	IFNG	62
	lymphotoxin B (LTB)	93
	chemokines XCL1/2	135
	chemokines (CCL, CXCL), IL16, oncostatin M (OSM), TGFB1	15–20, 35–41, 77, 106, 125
all	MIF	97
	S100A4/6/10/11	118
	VEGFA	133

Furthermore, a number of ligand mRNAs and several receptor mRNAs are strongly expressed by all three cell types (Table II). Moreover, numerous receptors were found to be expressed at a high level in at least one cell type, whereas neither cell type expressed the cognate ligands (Fig. 5*B*). These include adiponectin receptors (ADIPOR), CSF2R, CSF3R, IL2R, IL17R and the insulin receptor (INSR) (Fig. 5*B*: group IDs 4, 30, 31, 65, 78, 88). This suggests that ligands produced by other cell types of the HGSOC microenvironment or systemic factors might activate these receptors. Conversely, we also detected expression of ligands without expression of their cognate receptors, *e.g.* KITLG (Fig. 5*B*: group ID 89). Moreover, multiple orphan ligands were highly expressed in TU, TAM, and TAT, including ligands with known protumorigenic effects, like SPARC, SPP1, and STC1/2 (supplemental Fig. S7).

We further extended our analysis to the potential contribution of lipid mediators to the TU - TAM - TAT signaling network (supplementary Data set S10*B*). Toward this end, we determined the mRNA expression levels of enzymes involved in (phospho)lipid breakdown or in the generation of fatty acids with signaling functions, as well as of their respective receptors (Fig. 5*C*; (supplementary Data set S17)). We found an overall similar contribution by all three cell types, but also identified cell type-selective pathways:

- All cell types seem to partake in the generation of all analyzed types of lipid mediators (based on the expression of the corresponding enzymes), with a slightly more prominent role for TAM in the synthesis of lysophosphatidic acid (LPA) and leukotrienes.

- Although TU express the LPA receptors LPAR1–3, LPAR6 is the main LPA receptor on both types of immune cells.

- Leukotriene receptors (4 subtypes) are mainly expressed by TAM and TAT.

- Likewise, prostaglandin receptors are mainly expressed by both types of immune cells with a very prominent role for the PGE_2_ receptors PTGER2 and 4.

Emerging evidence from clinical trials suggests a therapeutic benefit for immune checkpoint inhibitors in the treatment of ovarian cancer (41). We therefore investigated whether cells of the HGSOC microenvironment expressed molecular targets of clinically relevant immune checkpoint inhibitors. Indeed, we found high mRNA expression levels of several checkpoint regulators particularly on immune cells, including PD-L1 on TAM and TAT, and CTLA4 on TAT (Fig. 5*D*).

##### TAM from Patients with Predicted Opposite Clinical Outcomes Express Distinct Sets of Growth Factors and Cytokines

**Fig. 6.**
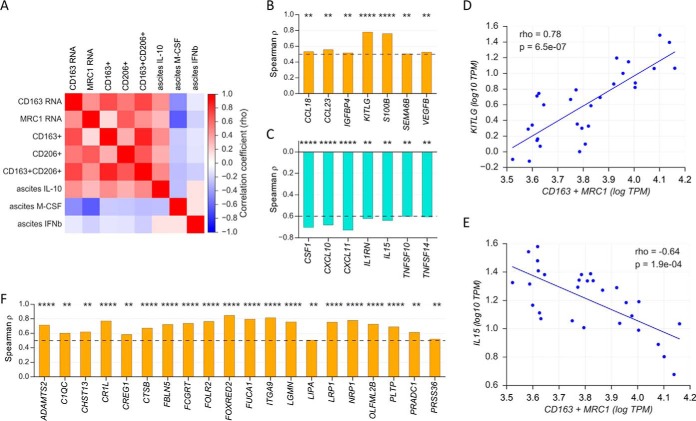
**Coexpression of genes coding for secreted proteins in TAM with surrogate markers of clinical outcome.** Both CD163 and MRC1 (CD206) are strongly associated with a poor clinical outcome (see main text). *A*, Heatmap illustrating the correlations (Spearman ρ) between CD163 and MRC1 (CD206) mRNA expression (RNA-Seq data), the frequency of CD163+ and CD206+ cells in fraction of CD14+ cells used for RNA-Seq (flow cytometry data) and cytokine levels (IL-10, M-CSF, IFNβ) in the corresponding ascites samples (ELISA). *B*, *C*, Spearman correlation analysis of *CD163* and *MRC1* mRNA levels (combined) in TAM with the expression of growth factor and cytokine genes. Panel (b) shows the positively correlated genes (ρ>0.5), panel (*C*) the inversely correlated genes (ρ<-0.6). Dashed lines indicate ρ values of 0.5 and −0.6 respectively. *D*, *E*, Scatter plots showing examples of correlations identified in panels (*B*) (*KITLG*) and (*C*) (*IL15*). The diagonals represent linear regression lines. A complete list of all correlating genes is shown in supplementary Data set S18. *F*, Analogous analysis as in (b), but for genes encoding secreted proteins other than growth factors or cytokines (top 15 genes). ****p < 0.0001; ***p < 0.001; **p < 0.01; *p < 0.05.

We have previously shown that TAM in ovarian cancer ascites can be stratified into subsets based on the expression of the surface markers CD163 and CD206 (encoded by the MRC1 gene), both of which show a high degree of variance among patients (1–94% CD163^+^ or CD206^+^ of the CD14^+^ TAM population) (8). Although a high abundance of these biomarkers is associated with a poor clinical outcome, a low expression is linked to longer relapse-free survival (RFS) (13–15). We have termed TAM of the former subgroup bTAM (“bad prognosis TAM”), and the low expression phenotype gTAM (“good prognosis TAM”). Consistent with these previous findings, we found strong correlations of *CD163* and *MRC1* mRNA levels with the fraction of CD163^+^/CD206^+^ cells in CD14^+^ TAM populations in our sample set (Fig. 6*A*). *CD163* and *MRC1* mRNA expression also correlated with the ascites levels of IL-10 (Fig. 6A), which is prognostic of a short RFS of ovarian cancer (30).

Based on this knowledge we next sought to investigate whether these different TAM subsets express distinct sets of growth factors and cytokines that might be relevant to the pathobiology of ovarian cancer. We applied Spearman correlation analysis to the genes coding for growth factors and cytokines (supplementary Data set S10*A*) in the transcriptomes of TAM to identify genes coexpressed with *CD163* and *MRC1*. Although 13 genes showed a significant positive correlation (supplementary Data set S18); top 7 in Fig. 6*B*), 28 were inversely correlated (supplementary Data set S6; top 7 in Fig. 6*C*). Gene ontology enrichment analysis identified the term “monocyte chemotaxis“ (GO:0002548) as the top hit for bTAM (fold enrichment >100; *p* < 0.0001), whereas “regulation of T-cell chemotaxis“ (GO:0010819) showed the highest score for gTAM (fold enrichment >100; *p* = 0.01). These correlations are in agreement with the clinically favorable intratumoral presence of T cells and the known unfavorable accumulation of monocytic cells in ovarian cancer (17, 42). This is also consistent with the higher expression of protumorigenic growth factors and cytokines by bTAM, *e.g. CCL18, KITLG, SEMA6B, S100B* and *VEGFB* (Fig. 6*B*). In contrast, tumor suppressive mediators made up most inversely correlated genes, *e.g. CXCL10, CXCL11, IL15, TNFSF10/TRAIL*, and *TNFSF14/LIGHT* (Fig. 6*C*). Examples of scatter plots for both directly (*KITLG*) and inversely (*IL15*) correlating genes are shown in Figs. 6*D* and 6*E*.

We also performed an analogous analysis for genes encoding secreted proteins other than growth factors and cytokines, and identified a set of 30 genes, whose mRNA expression was up-regulated in bTAM ((supplementary Data set S18); of these *n* = 20 with ρ>0.5; Fig. 6*F*). Predominant members of this group were proteins involved in ECM remodeling (*ADAMTS2, CTSB, FBLN5* (fibulin 5), and complement factors *C1QC* and *CR1L*).

##### Identification of Signaling Pathways in TU Associated with Clinical Outcome

**Fig. 7.**
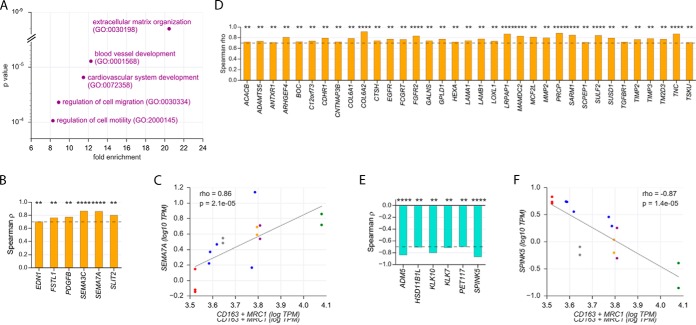
**Coexpression of genes coding for secreted proteins in TU with surrogate markers of clinical outcome.** *A*, The mRNA expression levels of genes encoding secreted proteins in TU were correlated to *CD163* and *MRC1* mRNA expression levels in TAM. Those genes showing a positive correlation (Spearman ρ>0.7) were functionally annotated (PANTHER). The figure shows the top non-redundant terms by enrichment (>8-fold) and *p* value (<0.001). *B*, Spearman correlation analysis of *CD163* and *MRC1* mRNA levels (combined) in TAM with the expression of growth factor and cytokine genes in TU (ρ>0.7). *C*, Scatter plot showing the correlation of *CD163* and *MRC1* mRNA levels in TAM (combined) with *SEMA7A* mRNA expression in TU. *D*, Analysis as in (*b*) for genes encoding secreted proteins other than growth factor or cytokine ligands. *E*, Analysis as in (*b*) for inversely correlated genes (ρ<-0.7). *F*, Scatter plot showing the inverse correlation of *CD163* and *MRC1* mRNA levels in TAM (combined) with *SPINK5* mRNA expression in TU. *C*, *F*, The diagonals represent linear regression lines. ****p < 0.0001; ***p < 0.001; **p < 0.01; *p < 0.05. Blue dots represent spheroids from different patients. Other colors represent patients with more than one type of spheroids, differing in ploidy and/or size (identical colors indicate matched samples from the same patient). A complete list of all correlating genes is shown in supplementary Data set S19.

A similar approach was used to ask whether the expression of genes encoding secreted proteins by TU differs among patients with opposite clinical outcomes (as predicted from the phenotype of their TAM). Toward this goal, we analyzed the transcriptomes of matched pairs of TU -TAM samples from 10 patients (Supplemental Table S1). Spearman correlation analyses identified 41 genes coding for secreted proteins (ρ>0.7; Supplementary Data set 19), the expression of which correlated with the presence of bTAM. GO enrichment analysis revealed ECM organization, vasculogenesis and cell migration/motility as functions associated with these genes (Fig. 7*A*). Of these genes, 6 coded for growth factors/cytokines (Figs. 7*B* and 7*C*) and 35 for other secreted proteins (Fig. 7*D*). Intriguingly, 3 out of the 6 proteins of the former group represent axon guidance molecules (SEMA3C, SEMA7A, and SLIT2). In addition, we found 6 inversely correlated genes coding for secreted proteins (ρ<-0.7; Figs. 7*E* and 7*F*), none of which belonged to the growth factor/cytokine group. Of these 6 genes, 3 coded for proteases or protease inhibitors related to kallikrein (KLK7, KLK10, SPINK5).

In our effort to unravel prognostic factors expressed by TU, we also made use of the PRECOG database, which contains the results of a meta-analysis of 1763 patients from 12 studies associating gene expression in ovarian tumor tissue with overall survival (OS) (28). Genes with a z-score of >4.0 or <-4.0 were used as surrogate markers for a correlation-based coexpression analysis, which resulted in the definition of 16 groups (supplemental Fig. S8*A*; (supplementary Data set S20)). Two groups clearly stood out: the PALLD group strongly associated with a short OS (Supplemental Fig. S8*B*), and the HLA-DQB1 group linked to a favorable clinical outcome (Supplemental Fig. S8*C*).

## DISCUSSION

A recently published proteogenomic study of HGSOC solid tumor tissue (36) found 9170 proteins associated with gene names (HGNC symbols), of which 6589 overlap with the combined proteomes of TU, TAM, and TAT in the peritoneal HGSOC microenvironment as reported here (supplemental Fig. S9). Conversely, 597 of the 7186 proteins in the HGSOC microenvironment proteome were not present in the tumor tissue proteome (supplemental Fig. S9). These differences between solid tumor tissue and peritoneal tumor microenvironment proteomes are presumably partially because of differences in the cellular composition (*e.g.* additional host cell types in solid tumor tissue), but also suggest that these two compartments differ significantly with respect to the functional properties of cells (*e.g.* detached *versus* attached tumor cells) as well as to the signaling networks operating among the different cell types. Therefore, these differences may be of high relevance for HGSOC biology and clinical treatment of the disease.

Our proteotranscriptomic analysis of different cell populations in the HGSOC microenvironment allows for the construction of intercellular communication maps. Although our study confirms published data on signaling pathways in HGSOC (5), it also uncovers signaling networks, which had not been recognized to operate in HGSOC and to be associated with clinical outcome so far.

**Fig. 8.**
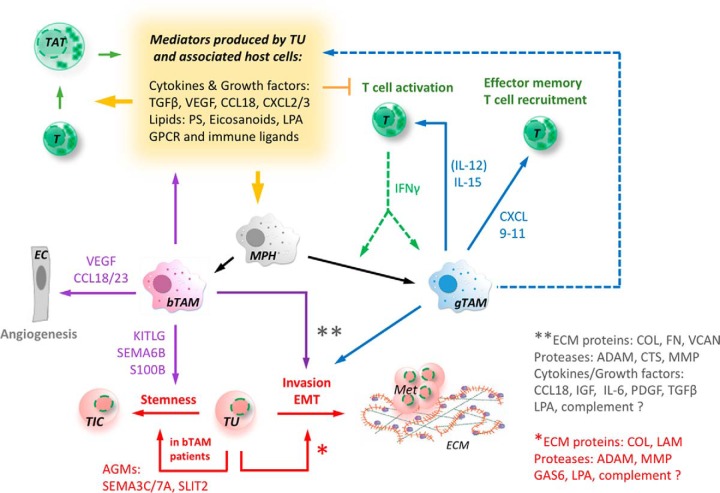
**Schematic representation of key intercellular communication pathways in HGSOC ascites.** gTAM (CD163^low^) produce factors that recruit and activate T cells (CXCL9–11, IL-15), consistent with their association with a favorable clinical outcome (15) and the up-regulation of interferon signaling in gTAM(99), possibly triggered by IFNγ from T cells. In contrast, bTAM (CD163^high^) secrete proteins promoting cancer cell stemness and angiogenesis, in line with the association of bTAM with a short survival. Cancer cells in patients with bTAM produce additional stemness-promoting factors (SEMA, SLIT). Both, gTAM and bTAM, as well as TU, produce a plethora of additional factors that reprogram immune cells to be immunosuppressed, immunosuppressive and protumorigenic. The latter include partly cell type-selective sets of ECM remodeling proteins secreted by TU and TAM. EC: endothelial cell; MPH: macrophage; T: T cell; TIC: tumor-initiating cell; AGM: axon guidance molecules; Met: metastasis; ECM: extracellular matrix.

A conspicuous outcome of our proteotranscriptomic analyses is the prominent expression of proteins involved in adhesion and ECM remodeling (schematic summary in Fig. 8 and supplemental Fig. S10*A*). These findings are in line with multiple reports showing that ovarian cancer cell metastasis depends on adhesion to mesothelial cells, invasion through the mesothelial cell layer, as well as remodeling of the submesothelial matrix (5, 43, 44), and that expression of ECM remodeling genes is associated with an unfavorable clinical outcome (45–50). The present study identifies members of the PALLD group, which play a role in cell adhesion, motility and ECM interactions (51, 52) to be expressed in TU and to be coexpressed with surrogate markers of a short RFS. Furthermore, our data show that TU and TAM play a major role in the synthesis both of ECM proteins such as collagens, laminins, fibronectin (FN), and versican (VCAN), as well as of ECM-degrading proteineases and their inhibitors including matrix metalloproteinases (MMPs), SERPIN and TIMP proteinase inhibitors. Our findings also suggest that TU and TAM impinge on ECM-associated processes in a cell type-selective fashion. Although laminins, for example, are predominantly produced by TU, most collagens are secreted mainly by TAM, which are also the major source of most proteinase inhibitors. Intriguingly, these findings suggest that TU and TAM cooperate in restructuring the ECM, cancer cell adhesion and invasion. This concept is in line with previous reports demonstrating a critical role for macrophages in ECM remodeling and cancer cell invasion of ovarian carcinoma (8, 15, 42) and other tumor types (53–56) reviewed by (11).

Our study also identified several functionally different classes of immunosuppressive mediators operating in the HGSOC microenvironment. These mediators are frequently produced by distinct cell types and act on specific types of immune cells, as schematically summarized in Fig. 8 and supplemental Fig. S10*B*. Besides TAM, TAT are a major source of many immunosuppressive factors, which we believe results from Treg within the TAT population, a conclusion that needs substantiation in future studies. The major players within the immune regulatory network in HGSOC ascites as revealed in this study are:

### 

#### 

##### - Cytokines

Cytokines play a major role in immunosuppression in the HGSOC microenvironment (57). Examples are TGFβ and VEGF secreted by all cell types; LIF mainly by TAT; and IL-10 selectively by TAM. These cytokines are well-known to skew the differentiation, polarization and/or function of both innate and adaptive immune cells toward an anti-inflammatory or regulatory phenotype lacking antitumor activity (57).

##### - Chemokines

CCL28 is synthesized mainly by TAT; CCL5, CXCL8, IL1RN by TAT and TAM; and CCL18, CXCL2, CXCL3 selectively by TAM. The function of most of these molecules is attraction of monocytes/macrophages and resting cells rather than effector T cells, resulting in tumor promotion by TAM and the lack of a cytotoxic antitumor response (58).

##### - Lipid Mediators

PGE_2_, a known T cell-suppressive eicosanoid (59), is produced by all analyzed cell types via PTGS (COX) and PTGES synthases. LPA, however, is produced mainly by TAM, which are also the major sources or phospholipases and autotaxin. Besides its role in cancer cell invasion (60), LPA has less well understood roles in macrophage differentiation (61) and T-cell function (62, 63).

##### - Checkpoint Regulators

Immune checkpoints play a decisive role in the blockade of cancer cell-directed T-cell responses (41). Our data show that TAT are a major source of checkpoint regulators: PD-L1 is derived from both TAM and TAT, whereas CTLA4 and LAG3 are selectively synthesized by TAT.

##### - Immunoligands

The proteases ADAM10, ADAM17 and PDI6, produced by all cell types, mediate the shedding of membrane-bound immunoligands (*e.g.* MICA, MICB), thereby producing molecules that inhibit stimulatory receptors on NK and T cells (*e.g.* NKG2D) (64, 65).

Intriguingly, our analyses uncovered an extensive signaling network of axon guidance molecules (AGM) of the ephrin (EFN), semaphorin (SEMA), and slit (SLIT) families and their cognate receptors, Eph (EPHA, EPHB), plexins (PLXN), neuropilins (NRP), and robo (ROBO). Although there is plenty of evidence that these axon guidance molecules play important roles in the microenvironment of multiple cancer types (66–69), very little is known about their potential functions in ovarian cancer. Our data show that Slit2 is selectively expressed in TU, and that this expression correlates with the bTAM phenotype. Interestingly, it has been shown that Slit2 exerts inhibitory activity on chemokine-induced migration of lymphocytes, and that this effect is mediated by Robo1 (70, 71), which we find to be expressed on all cell types of the HGSOC microenvironment. In colorectal carcinoma cells, Slit2/Robo1 signaling promotes degradation of E-cadherin, EMT, tumor growth and metastasis (72). Of note, SLIT2 also stimulates angiogenesis via Robo1 and Robo2 in the mouse retina (73). Among the semaphorins detected in TU, we found Sema3C and Sema7A to be associated with surrogate markers of adverse patient prognosis. In line with this, Sema3C has been reported to promote survival and tumorigenicity of glioma stem cells in an autocrine/paracrine manner (74), and to enhance adhesion, migration and invasion of breast cancer cells (75, 76). Additionally, tumor-promoting functions have been described for Sema7A in breast cancer. Mechanistically, Sema7A seems to act both on tumor cells to increase invasion as well as on TAM to induce the release of proangiogenic molecules (77, 78). Overall, our study implicates an AGM network in central processes of ovarian cancer biology.

A striking feature of our proteomic data is the presence of virtually the complete complement system, including all secreted classical complement proteins (C1-C9 with C5 only in secretome) and 9 out of 10 complement receptors (CR2 not found in the receptome). Furthermore, multiple proteins functioning as complement regulatory factors or involved in non-classical complement activation were readily detectable, such as complement factors B, H, I, and P as well as ficollins 1, 2, and 3. TAM play a prominent role within this network as the major producers of most soluble factors and receptors. These observations are intriguing in view of a large body of evidence implicating the complement system in cancer growth and progression, apart from its canonical role in immune defense (79–84). Complement components have been demonstrated to promote angiogenesis, cell proliferation, cellular survival, extracellular matrix degradation, tumor cell invasion and migration (84), and chemotaxis of mesenchymal stem cells into the tumor microenvironment (85). Moreover, complement components interfere with cancer immune surveillance. Despite the detection of all soluble complement components in the HGSOC microenvironment, our data suggest that tumor cells are spared from complement attack because of the presence of inhibitory receptors and membrane-bound complement regulatory proteins, such as CR1, CD46, CD55, and CD59, which, however, have been shown to not block the protumorigenic effects of complement (86).

As the sample sizes of our proteotranscriptomic study do not allow for the analysis of potential associations with clinical outcome, we sought to address this issue by using surrogate markers of RFS. The only established prognostic markers for cells in ovarian cancer ascites are the surface expression of CD163 and CD206 in TAM (13–15). By applying Spearman correlation analysis, we were able to identify sets of protumorigenic genes in bTAM and of tumor-suppressive genes in gTAM (Fig. 8 and supplemental Fig. S11). Our data show that bTAM preferentially express growth factors and cytokines known to promote ovarian cancer growth, progression and relapse, such as CCL18, but also factors, which have previously not been implicated in ovarian cancer progression, *e.g.* KITLG and complement factors. CCL18 levels in tumor tissue are associated with metastatic spread and a shorter survival in ovarian cancer patients, which appears to involve an increase in mTORC2 signaling (87). Although this study, however, described expression by tumor cells, a previous report concluded that CCL18 was selectively expressed by tumor-associated host cells with macrophage morphology (88). Our own data (Fig. 5*A*) clearly support the latter observation. CCL18 has also been linked to pancreatic carcinoma, where it promotes EMT and cancer cell invasion (89), processes that are also instrumental in ovarian cancer spread. Furthermore, CCL18 secreted by TAM has previously been reported to promote angiogenesis and metastasis formation in breast cancer (90, 91), consistent with our findings for HGSOC. The selective expression of KITLG by bTAM is intriguing in view of its function as a stemness-promoting factor and the previous identification of its receptor CD133 as a marker for ovarian epithelial stem cells in the mouse (92) and for cancer stem cells in ascites (93, 94). CD133 expression has also been shown to promote ovarian cancer metastasis and resistance (92, 95, 96), and is associated with a worse clinical outcome (97). Moreover, bTAM also overexpressed a number of proteins associated with the ECM or its restructuring, including ECM degrading proteases, suggesting that bTAM play a particular role in assisting cancer cell adhesion and invasion.

GO enrichment analysis showed a strong association of the gTAM expression signature with “regulation of T-cell chemotaxis.” Mediators of relevance in this context are the chemokines CXCL9, 10 and 11, a major effect of which is the chemoattraction of effector memory T cells. This is consistent with the known strong association of a favorable clinical outcome of ovarian cancer with the intratumoral presence of T cells (17), the clinical benefit of a PD1 checkpoint blockade in a subset of ovarian cancer patients (98), the up-regulation of IFN signaling in CD163+ TAM (99), and the observed preferential synthesis of IL-15 by gTAM (Figs. 6*C*, 6*E*, 8 and supplemental Fig. S11). IL-15 promotes the effector functions of both CD8+ T cells and natural killer (NK) cells and thus plays a pivotal role in cancer immunosurveillance (100, 101). Our data also reveal that gTAM express higher levels of LIGHT (TNFSF14), a TNF superfamily member that interacts with tumor-associated myeloid cells, NK cells, T cells and tumor cells through its receptors, CD270 and lymphotoxin β, to augment the recruitment, retention and activation of effector cells, resulting in strong antitumor responses (102). Another TNF superfamily member found to be synthesized selectively by gTAM is TRAIL (TNFSF10), which is known to have direct suppressive effects on tumor cells by inducing cell death (103). Taken together, our findings clearly suggest that bTAM and gTAM have opposing functions in ovarian cancer progression, with bTAM promoting tumor cell adhesion, invasion, survival and stemness, and gTAM contributing to a better clinical outcome by attracting and enhancing the activation of cytotoxic immune cells.

In this context, it is important to note that antibody or small molecule drugs inhibiting the functions of many of the (potentially) relevant signaling axes identified in the present study already exist. These include pathways and mechanisms not yet clinically addressed for HGSOC. This raises the possibility to achieve a rapid clinical translation for pathways that prove to be functionally relevant in follow-up studies of the present work. Furthermore, multiple pathways identified in this study are driven by ligand-receptor interactions and/or ligand-generating enzymatic reactions, and thus are amenable to pharmacological interference by future drugs.

This first proteotranscriptomic characterization of the ovarian cancer microenvironment will serve as a public resource and provide a framework for further functional analyses. Our study identifies intercellular communication networks in the ovarian cancer microenvironment, and uncovers associations of cell type-specific expression signatures with clinical outcome.

## DATA AVAILABILITY

RNA-Seq data were deposited at EBI Array-Express (accession numbers E-MTAB-3167, E-MTAB-4162, E-MTAB-5199, E-MTAB-5498). Protein mass spectrometry data were deposited at PRIDE Accession No. PXD006138 (supplemental Table S1) and PXD008047 (proteomic analyses of conditioned media of TU and TAM). Data can also be accessed via MS-viewer (http://msviewer.ucsf.edu/prospector/cgi-bin/msform.cgi?form_msviewer), a web-based spectral viewer for proteomics results (31), by entering the search keys “tvk5oek0to” or “y40gjy2o1y” (proteomic analyses of conditioned media of TU and TAM).

## Supplementary Material

Supplemental Data

## Footnotes

^1^ The abbreviations used are:

HGSOC: high grade serous ovarian adenocarcinoma
bTAM: bad prognosis TAM
ECM: extracellular matrix
FDR: false discovery rate
GF: growth factor
GO: gene ontology
gTAM: good prognosis TAM
LFQ: label-free quantification
LPA: lysophosphatidic acid
MACS: magnetic cell sorting
PCA: principal component analysis
RNA-Seq: RNA sequencing
RFS: relapse-free survival
TAM: tumor-associated macrophage
MS: mass spectrometry
TAT: tumor-associated lymphocyte
TU: tumor cell spheroid
TME: tumor microenvironment
TPM: transcripts per million.
