# Supplementary material for: Proteotranscriptomics Reveal Signaling Networks in the Ovarian Cancer Microenvironment
Source: Mol Cell Proteomics. 2017 Nov 15;17(2):270–89. doi: 10.1074/mcp.RA117.000400 (PMC5795391; doi:10.1074/mcp.RA117.000400)
Supplement: Supplemental Data [file supp_17_2_270__index.html]

Proteotranscriptomics Reveal Signaling Networks in the Ovarian Cancer Microenvironment. — Proteotranscriptomics of ovarian cancer. — Proteotranscriptomics Reveal Signaling Networks in the Ovarian Cancer Microenvironment — Proteotranscriptomics of Ovarian Cancer — Supplemental Data 

# Proteotranscriptomics Reveal Signaling Networks in the Ovarian Cancer Microenvironment

## Supplemental Data

- Supplementary Material - Supplementary Material
- Supplementary Table 1 - Supplementary Table 1
- Supplementary Datasets - Supplementary Datasets
