# Supplementary material for: Proteotranscriptomics Reveal Signaling Networks in the Ovarian Cancer Microenvironment
Source: Mol Cell Proteomics. 2017 Nov 15;17(2):270–89. doi: 10.1074/mcp.RA117.000400 (PMC5795391; doi:10.1074/mcp.RA117.000400)
Supplement: Supplemental Data [file supp_RA117.000400_133125_0_supp_12699_sxq6mt.pdf]

Supplementary Material

Proteotranscriptomics Reveal Signaling Networks in the Ovarian Cancer Microenvironment

Thomas Worzfeld[1,2#*], Florian Finkernagel[3#], Silke Reinartz[4#], Anne Konzer[5], Till Adhikary[3], Andrea Nist[6], Thorsten Stiewe[6], Uwe Wagner[7], Mario Looso[8], Johannes Graumann[5] and Rolf Müller[3*]

Content

Suppl. Note: Pages 2-3
Suppl. Fig. 1: Analysis of the proteome of TU, TAM and TAT (Pages 4-5)
Suppl. Fig. 2: Comparison of transcriptomes and proteomes (Pages 6)
Suppl. Fig. 3: Analysis of growth factors/cytokines and their receptors in the transcriptomes and proteomes of TU, TAM and TAT (Page 7)
Suppl. Fig. 4: p-value estimation for growth factors/cytokines and the respective receptors (Page 8)
Suppl. Fig. 5: Correlation of mRNA and protein expression for growth factor/cytokine receptors (Page 9)
Suppl. Fig. 6: Correlation of mRNA and protein expression for growth factors/cytokines (Page 10)
Suppl. Fig. 7: mRNA expression of orphan ligands in the HGSOC microenvironment (Page 11)
Suppl. Fig. 8: Coexpression analysis for proteins associated with clinical outcome (Page 12)
Suppl. Fig. 9: Comparison between the proteomes of tumor cells from HGSOC-associated ascites and ovarian tumor tissue (Page 13)
Suppl. Fig.10: Schematic summary illustrating the cooperation of different cell types in the HGSOC microenvironment in mediating specific biological functions (Page 14)
Suppl. Fig.11: Scheme depicting the contrasting roles of distinct TAM subsets in HGSOC biology (Page 15)

Supplementary Note

A total of 7,186 proteins were detected in TU, TAM and TAT (FDR=0.01 on the peptide and protein level, and minimum of 1 unique peptide per protein group), of which 6,442 proteins showed a median label-free quantification (LFQ) intensity >50 million (corresponding to the 0.25 quantile of the combined proteome). All TU, TAM and TAT samples showed a purity of >90% by microscopic or flow cytometry analysis. However, when analyzing the expression levels of multiple cell type-selective markers in our proteomic data, we found a significant contamination of 3 TAT samples with tumor proteins (see Supplementary Fig. 1a and Materials and Methods for details). We attribute this apparent discrepancy to the much larger size and thus higher protein content of TU relative to TAT. These TAT samples (Supplementary Table 1) were therefore excluded from analyses of cell type-selective expression, which would be distorted by TU protein contamination (see Materials and Methods for details). Principal component analysis (PCA) of the combined proteomes yielded a clear separation of TU, TAM and TAT samples (Supplementary Fig. 1b), indicating that the proteomic data were suitable for further in-depth analysis. To further illustrate statistically significant cell type-specific expression of proteins in TAM versus TU, TAT versus TU and TAT versus TAM, volcano plots were generated (Supplementary Fig. 1c and Supplementary Dataset 2). The top 30 genes with the highest selectivity for either of the cell types are depicted in Supplementary Fig. 1d. Gene ontology (GO) enrichment analyses of (i) TAT-specific proteins identified terms specific for T cell function, of (ii) TAM-specific proteins showed terms associated with macrophage function, and of (iii) TU-specific proteins revealed terms associated with cell growth, thus corroborating the validity of the proteomic analysis (Supplementary Fig. 1e).

In total, 7,186 proteins were identified in the proteomes. 7,100 proteins were undetectable in spite of readily detectable mRNA expression (49.7% detectable at TPM>2), while converse cases were comparably rare (n=125; 0.9%; Supplementary Fig. 2a, upper left panel; proteins with undetected mRNAs are listed in Supplementary Dataset 22). Such a detection of a protein without detection of the respective mRNA could be due to detection failure in mRNA sequencing or due to false positive detection in protein mass spectrometry (FDR filtration employed in this study is 1% on both the peptide and protein level). Supplementary Fig. 2a shows that that the overlap between transcriptomes and proteomes was considerably bigger (52.3%) for intracellular proteins (see Materials and Methods) compared to membrane or secreted proteins (37.7% and 43.2%, respectively). To identify the cause for the relatively low percentage of membrane and secreted proteins in the proteomes, we investigated the relationship between mRNA expression and the detection of the corresponding proteins. Genes encoding growth factor/cytokine receptors or their respective ligands, which were not detected by our proteomic analysis in spite of high TPM values, are shown in Supplementary Fig. 2b. To address the issue of discrepancies between transcriptome and proteome in more detail, we used flow cytometry to examine the expression of several proteins, which were present in the transcriptome, but absent from the proteome of TAM. For

comparison, we included S100A8/A9 and TGFB1 (which were found in the proteome) as positive controls. All proteins analyzed were detected by flow cytometry, albeit in vastly variable fractions of cells (e.g., IL1B in nearly 100% of the cells, TGFBRIII in only 3%) (Supplementary Fig. 2c, 2d and 2e).

Next, we studied the correlation between transcriptome and proteome for genes encoding growth factor/cytokine receptors and their ligands in a cell type-specific manner. Our transcriptomic analysis revealed mRNA expression of 181 genes encoding receptors and 209 genes encoding ligands (Supplementary Fig. 3a and b, Supplementary Datasets 12 and 13; for a list of the 76 receptors and 257 ligands not expressed in any of the cell types see Supplementary Dataset 14). Out of these expressed genes, 37 coding for growth factor/cytokine receptors (20.4%) and 44 coding for their ligands (22.0%) were also identified in the proteomes (Supplementary Fig. 3c and d). In the majority of cases, there was a positive correlation of mRNA and protein expression (Spearman ρ=0.3-1.0; Supplementary Fig. 5 and 6). Cell type-selective mRNA expression was higher for genes encoding ligands (n=74 expressed in all 3 cell types; 35.4%) than for genes encoding receptors (n=93; 51.4%).

Supplementary Figures

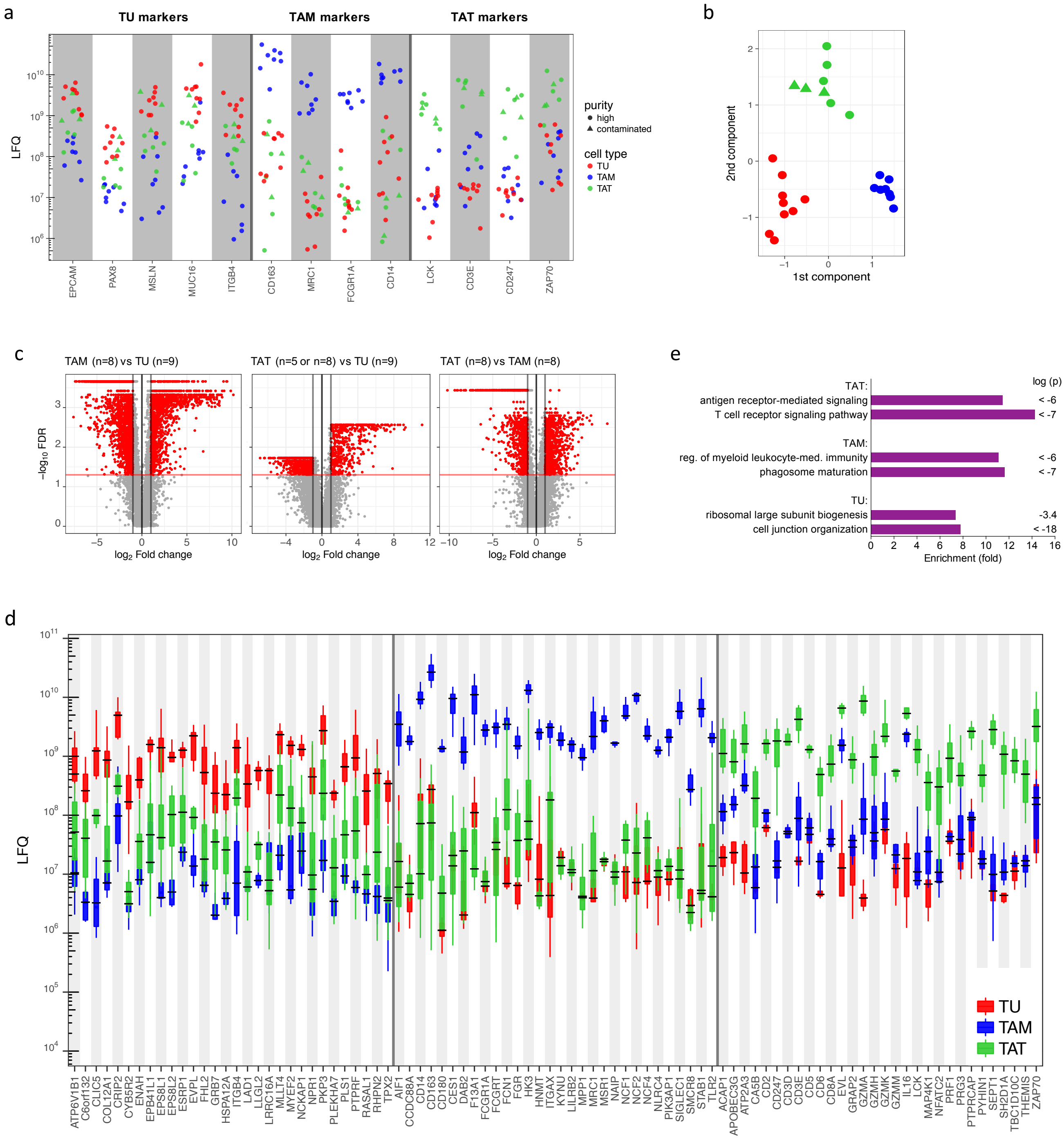

Supplementary Figure 1.

Analysis of the proteome of TU, TAM and TAT.

See next page for legend.

Legend to Supplementary Figure 1.

Analysis of the proteome of TU, TAM and TAT.

(**a**) Expression of cell type-specific marker proteins in tumor cells (TU, n=9), tumor-associated macrophages (TAM, n=8) and tumor-associated T cells (TAT, n=8). Expression levels are based on LFQ intensity values calculated from mass spectrometry data. Three TAT samples with significant (see Materials and Methods) levels of tumor marker expression are marked as "contaminated" (triangles). For these 3 TAT samples, the sum of LFQ intensities of tumor marker proteins was higher than in the TU sample with the lowest sum of LFQ intensities of tumor markers (see Materials and Methods for details). These samples were excluded from the analysis of proteins upregulated in TU versus TAT (panel (c) below; see Materials and Methods for details). (**b**) Principal component analysis (PCA) of the combined scaled proteomes of all TU, TAM and TAT samples. Color code and symbols as in panel (a). (**c**) Volcano plots analyzing cell type-specific expression of proteins in TAM versus TU, TAT versus TU, and TAT versus TAM. Statistically significant differences were identified by an unpaired permutation approach. False discovery rates (FDR) are plotted against fold difference calculated from median LFQ intensity values (red data points: false discovery rate <0.05; fold difference >2). Sample sizes are TAM vs TU: 8 vs 9; TAT vs TU: 5 (left half of second panel) or 8 (right half of second panel) vs 9; TAT vs TAM: 8 vs 8 (see Materials and Methods). (**d**) The top 30 (by LFQ intensity) cell type-selective (as determined in panel c) proteins are shown. Boxes: upper and lower quantile; horizontal lines: median; vertical lines: 95% confidence intervals. (**e**) PANTHER functional annotation (GO enrichment analysis) of cell type-specific protein sets identified in panel (c) (top 2 terms by enrichment and p-value).

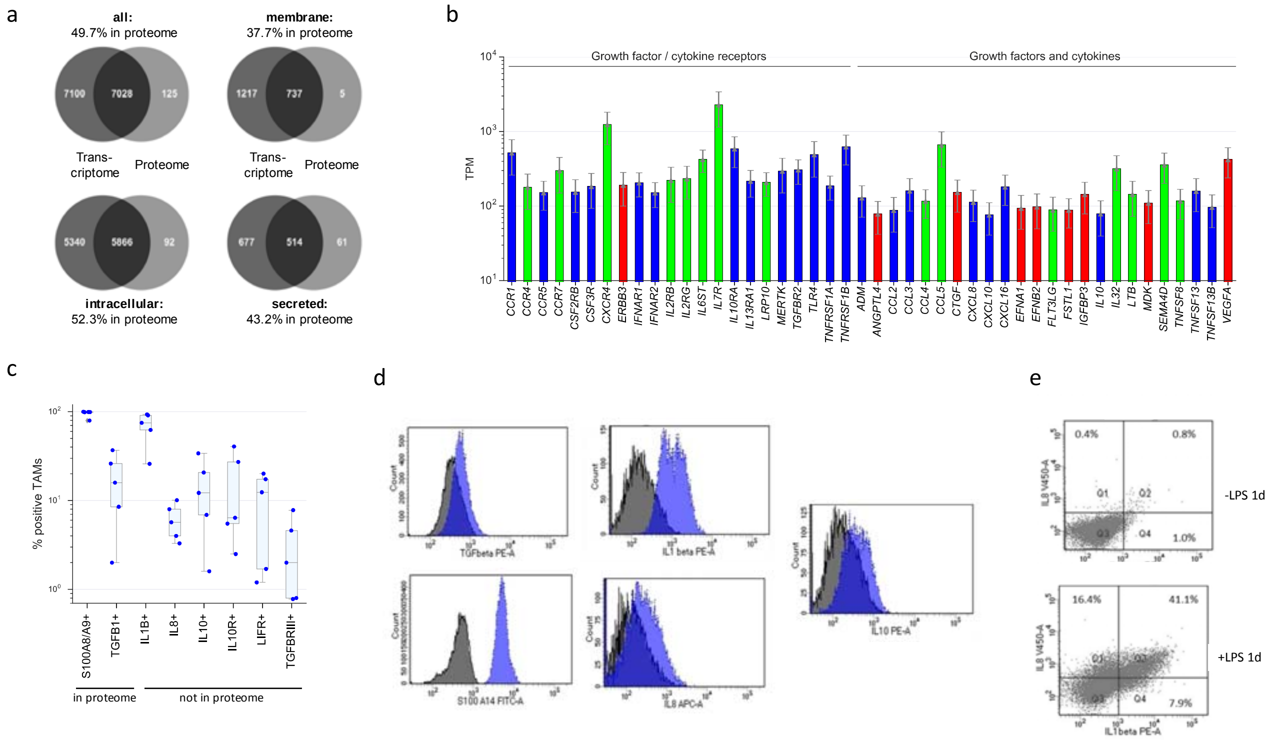

Supplementary Figure 2.

Comparison of transcriptomes and proteomes.

(**a**) Venn diagrams representing the number of detected mRNAs in transcriptomes (TPM>2) and proteins (with HGNC symbols) in proteomes of TU, TAM and TAT. The following sets of genes were analyzed: all genes (“all”); genes encoding secreted proteins (“secreted”) or membrane proteins (“membrane”), both sets derived from ProteinAtlas as described in Materials and Methods; and genes not in the latter two groups (“intracellular”). Some genes (n=306) were assigned to both the „secreted" and “membrane” set. (**b**) mRNA expression (TPM) of receptors and growth factors/cytokines, which have not been detected in the proteome. For each receptor and growth factor/cytokine, the expression value of the cell type with the highest median mRNA expression (TPM) is depicted (red: TU; blue: TAM; green: TAT; median ± STD). Only receptors and growth factors/cytokines with high mRNA expression (TPM>120 for receptor genes; TPM>60 for growth factor/cytokine genes) are shown. (**c**) Flow cytometry analysis of intracellular cytokines and membrane receptors in TAM of 5 patients. Boxes represent the median, and error bars show the standard deviation. IL1B, IL8 (CXCL8), IL10, IL10R, LIFR and TGFBRIII were not detected in the proteome, in contrast to S100A8/A9 and TGFB1 (Supplementary Dataset 1; see also Fig. 1d), which were included in the flow cytometry analysis for comparison. (**d**) Histograms of flow cytometric analyses of intracellular cytokine levels (IL-1β, IL-8/CXCL8, IL-10, S100A14 and TGFβ) in TAM (representative examples of data in panel c). Blue: specific antibodies; grey: matched isotype controls. (**e**) Dot plots showing the intracellular expression of IL-8 versus IL-1β in macrophages treated with solvent only (top) or with LPS for 24 hours (bottom). The experiment was carried out as a positive control.

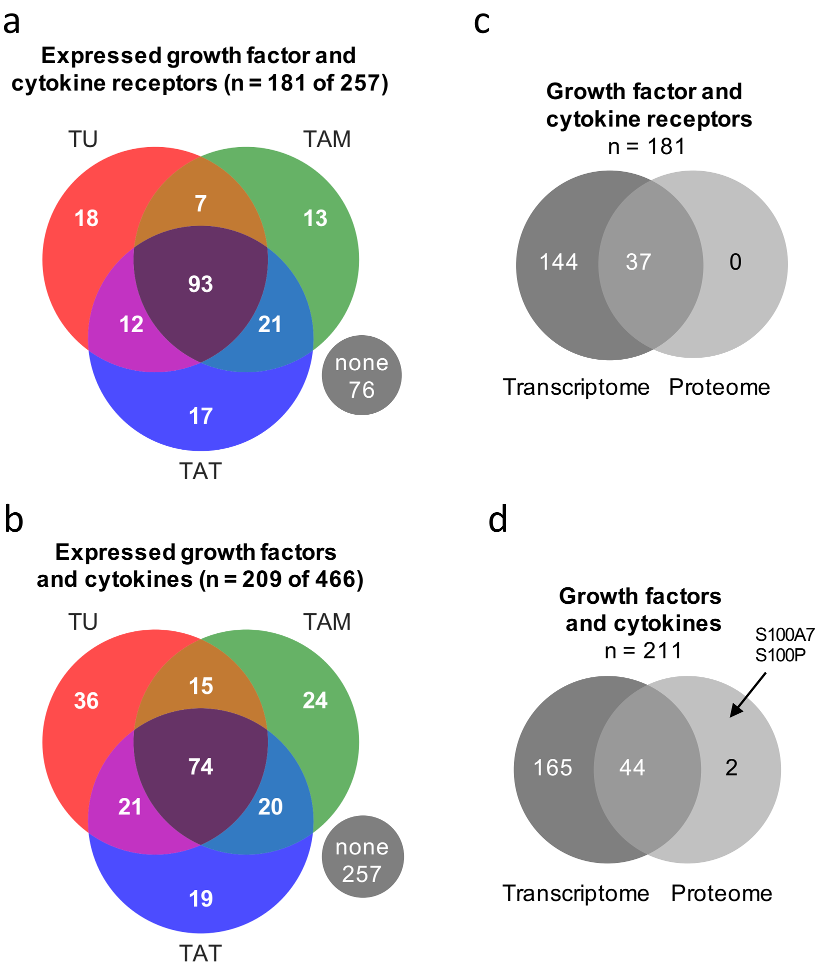

Supplementary Figure 3.

Analysis of growth factors/cytokines and their receptors in the transcriptomes and proteomes of TU, TAM and TAT. (**a**) Venn diagrams showing the number of growth factor/cytokine receptor genes expressed on the mRNA level in TU, TAM or TAT (TPM >2). (**b**) The analogous Venn diagram for growth factor/cytokines ("ligands"). (**c, d**) Venn diagrams illustrating the number of receptors (c) and growth factors/cytokines (d) in transcriptomes and/or proteomes (all cell types combined).

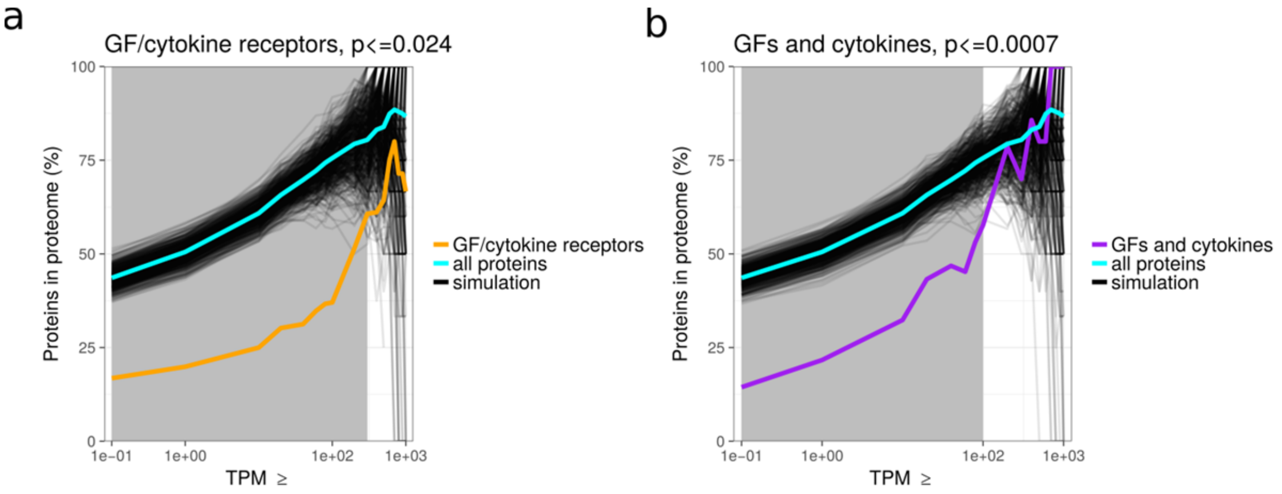

Supplementary Figure 4.

p-value estimation for growth factors/cytokines and the respective receptors. Visualization of the p-value estimation for growth factors/cytokine receptors (**a**) and their respective ligands (**b**) defined in Supplementary Dataset 10A in Fig. 1c. Ten thousand random sets of the same size were drawn from all protein-mRNA pairs to establish a p-value (only regions with at least 20 genes in the query (shaded grey) were considered). This figure shows a subsampling of the random sets (n=600). See Materials and Methods for details.

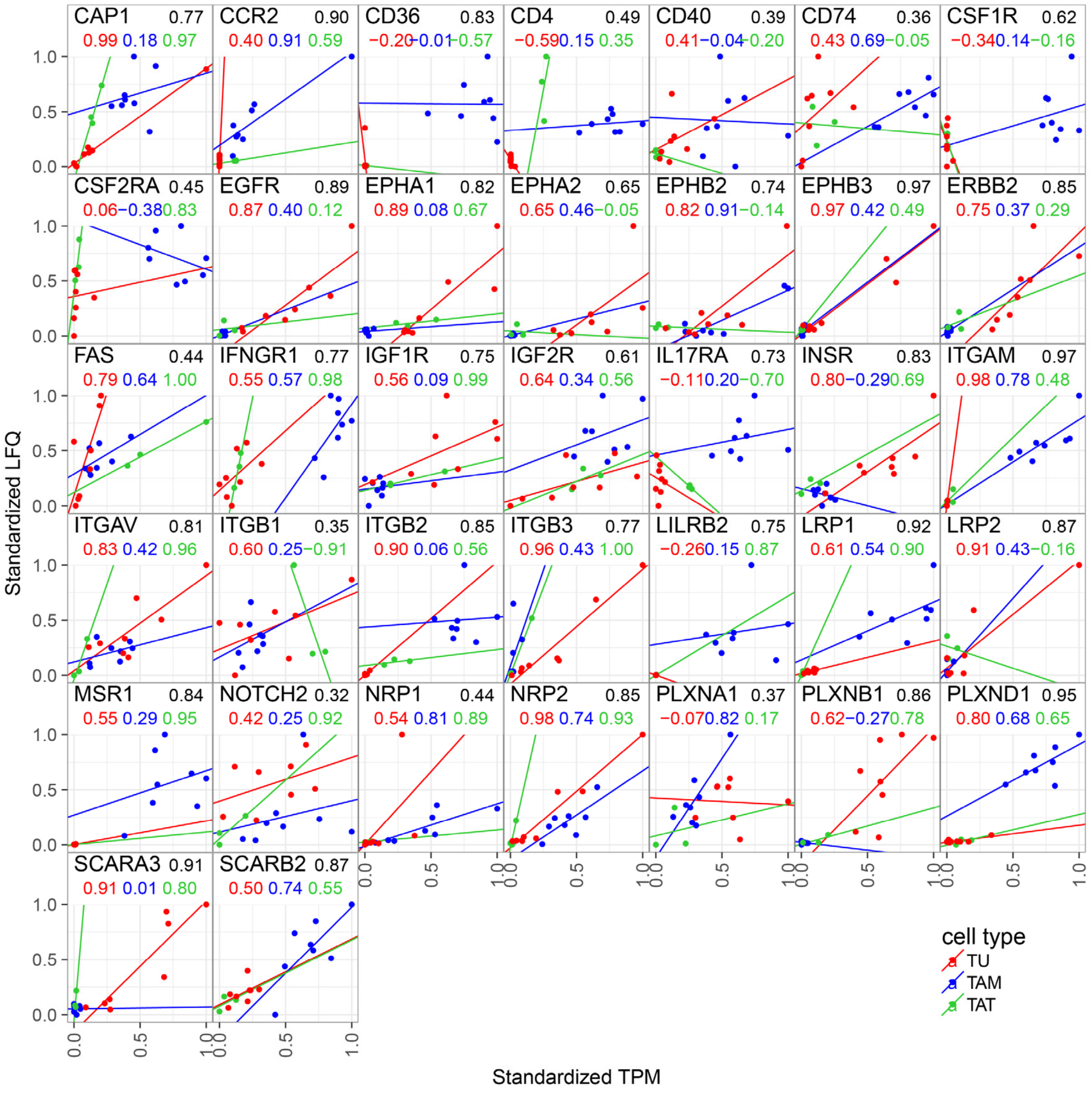

Supplementary Figure 5.

Correlation of mRNA and protein expression for growth factor/cytokine receptors. Standardized LFQ intensity values are plotted against standardized TPM values. Numbers indicate Spearman correlation ρ values. Each dot represents a different sample (patient). TU: red; TAM: blue; TAT: green.

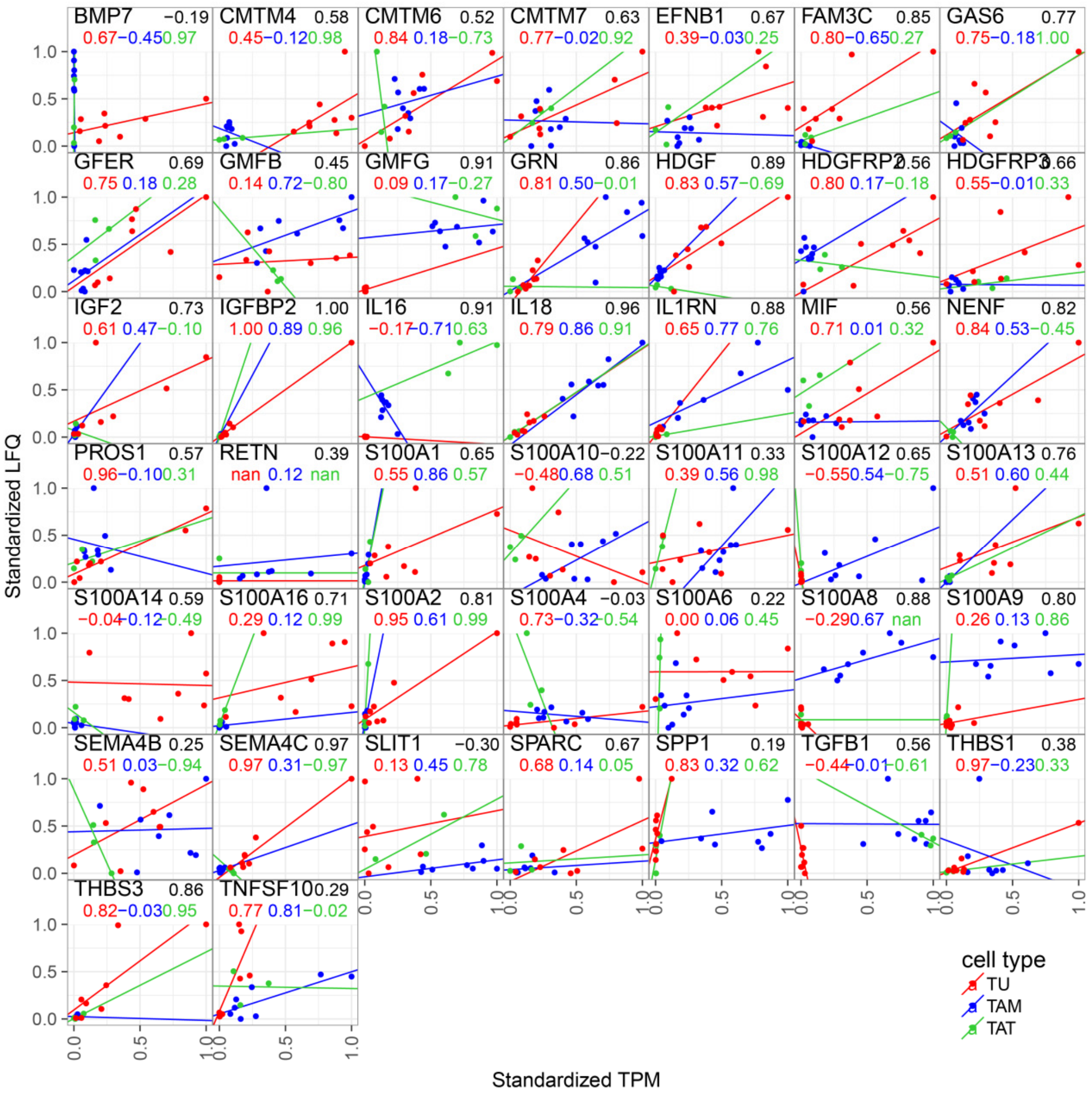

Supplementary Figure 6.

Correlation of mRNA and protein expression for growth factors/cytokines. Standardized LFQ intensity values are plotted against standardized TPM values. Numbers indicate Spearman correlation ρ values. Each dot represents a different sample (patient). TU: red; TAM: blue; TAT: green.

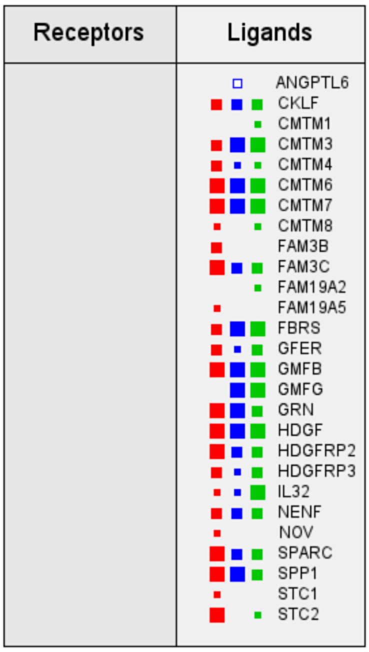

Supplementary Figure 7.

mRNA expression of orphan ligands in the HGSOC microenvironment. Transcriptomic analysis of genes encoding secreted proteins with functions in growth factor/cytokine signaling without known receptors ("orphan ligands"). The sizes of the filled squares for TU (red), TAM (blue) and TAT (green) indicate the level of expression determined by RNA-Seq (high: median TPM >50; intermediate: TPM >10; low: TPM >2). Open squares indicate cases, where substantial expression (TPM >3) was observed only in a small fraction of samples (<10%).

a

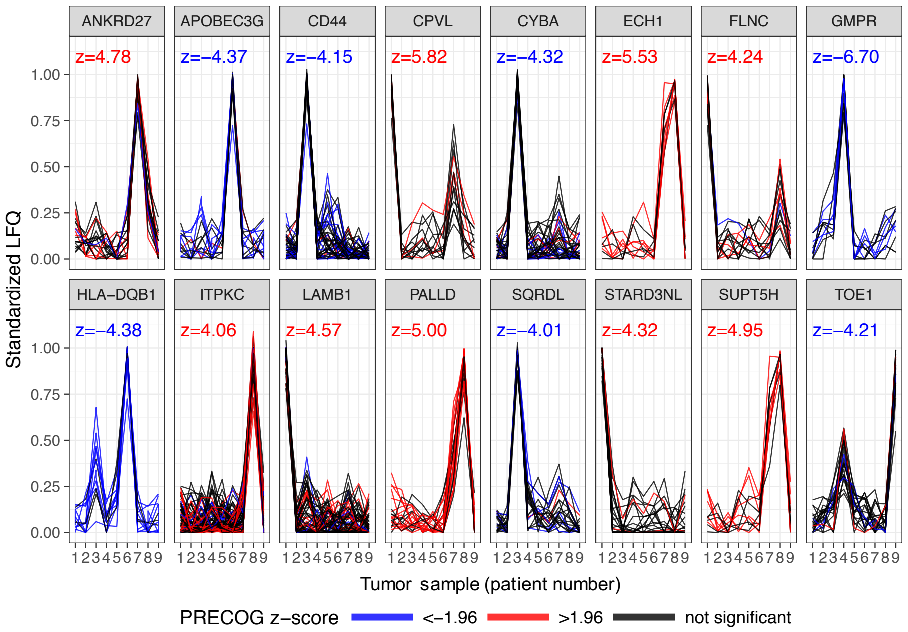

b

Protein	Description	z-score	Spearman ρ	TPM TU
ACTN4	actinin, alpha 4	3.9	0. 88	688
ADCK4	aarF domain containing kinase 4	3.0	0.95	34
ANXA4	annexin A4	2.7	0.91	64
NFKBIB	NFkB inibitor beta	3.2	0.45	13
PAK4	p21 (RAC1) protein-activated kinase 4	3.9	0.84	40
PALLD	palladin, cytoskeletal associated protein	5.0	0.33	42
PLD3	phospholipase D family, member 3	1.4	0.93	94
PPIC	peptidylprolyl isomerase C	4.0	0.96	29
SAMD4B	sterile alpha motif domain containing 4B	2.3	0.84	77
SARS2	seryl-tRNA synthetase 2, mitochondrial	4.2	0.79	25
SERPINH1	serpin peptidase inhibitor H1	2.1	0.88	168
SIPA1L3	signal-induced proliferation-associated 1L3	2.2	0.73	74
SIRT2	sirtuin 2	2.9	0.73	38

c

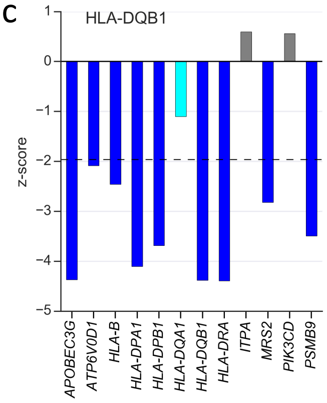

Supplementary Figure 8.

Coexpression analysis for proteins associated with clinical outcome. (**a**) The expression levels of proteins (LFQ intensities standardized to the maximum in each panel) in TU of 9 different patients is shown. Coexpression analysis was carried out for surrogate markers associated with a poor or favorable overall survival (OS) (PRECOG z-score >4 or <-4; details in Materials and Methods). The association of individual coexpressed proteins with OS is shown in red (poor OS), blue (favorable OS) or black (not significant). (**b**) Detailed results for proteins of the PALLD group with a Spearman correlation of RNA and protein expression of ρ>0.3. (**c**) PRECOG z-scores for proteins of the HLA-DQB1 group.

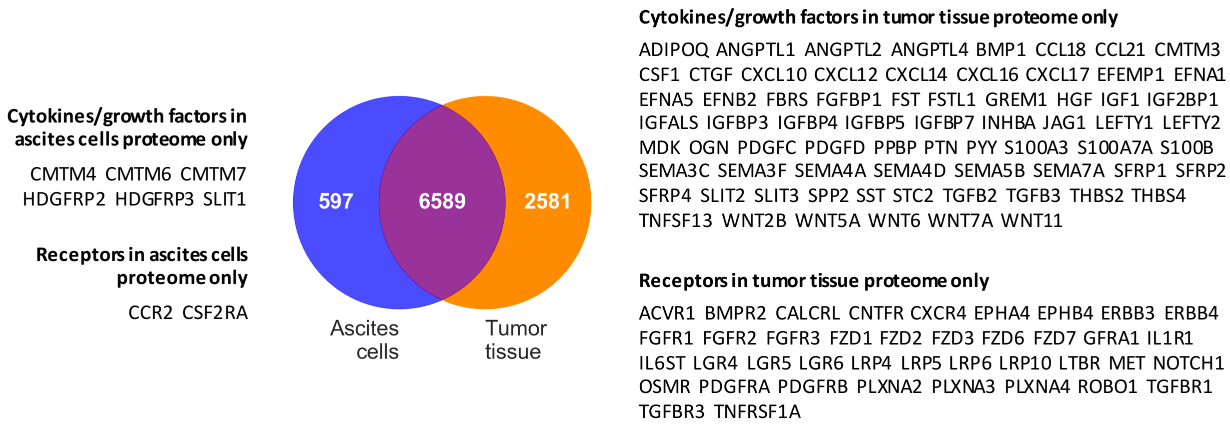

Supplementary Figure 9.

Comparison between the proteomes of tumor cells from HGSOC-associated ascites and ovarian tumor tissue. The combined proteome of TU, TAM, and TAT from ovarian cancer ascites (data from this study) was compared to the proteome of solid ovarian tumor tissue (Zhang et al, 2016). This analysis yielded an overlap of 6,589 proteins. 597 proteins were exclusively found in cells isolated from ascites, while 2,581 proteins were exclusively found in solid ovarian tumor tissue. Those growth factors/cytokines and their respective receptors, which were detected in one of the two proteomes only (but not in both), are listed.

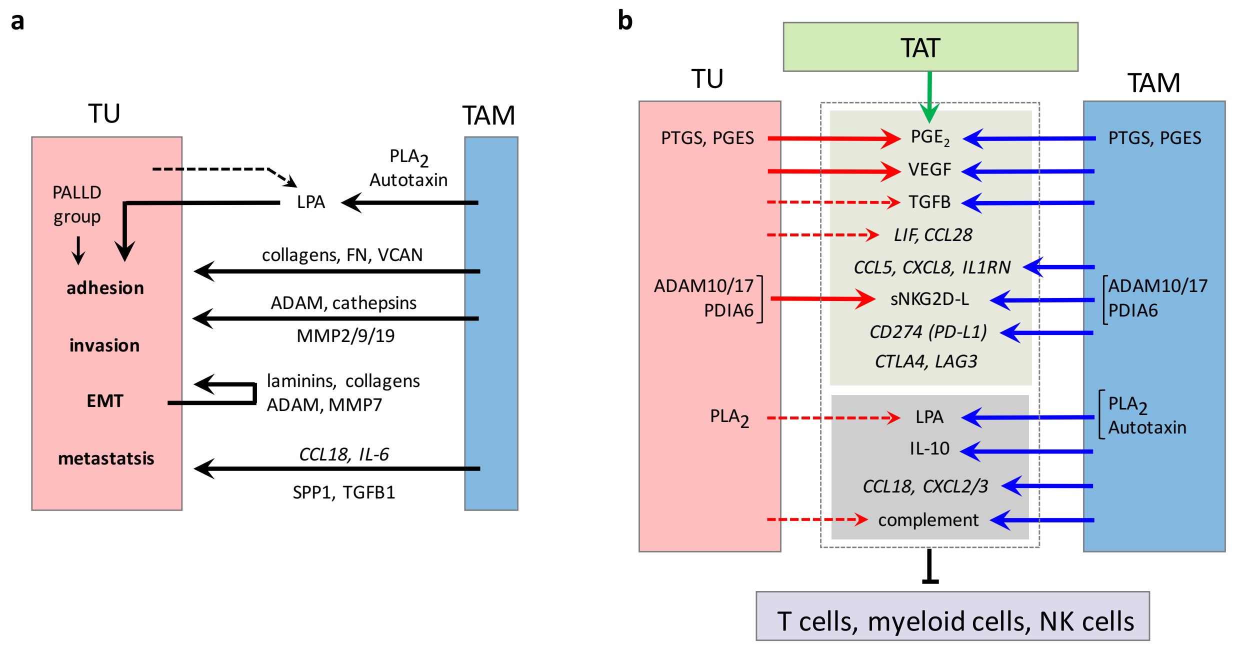

Supplementary Figure 10.

Schematic summary illustrating the cooperation of different cell types in the HGSOC microenvironment in mediating specific biological functions. (**a**) Cooperation of TU and TAM in invasion and metastasis. (**b**) Cooperation of TU, TAM and TAT in immune suppression. The schemes are based on the data in Figs. 2-5 and include pathways known to be associated with immune suppression or cancer cell invasion and metastasis. The box in the center (dashed outline) depicts mediators secreted by TU, TAM and/or TAT, or generated by the indicated enzymes (prostaglandin synthases, proteases, phosholipases). TAT synthesize the mediators listed in the upper box. Contributions by other cell types are shown by arrows. Proteins predicted from RNA-Seq data are italicized. The PALLD group is defined in Supplementary Fig. 8.

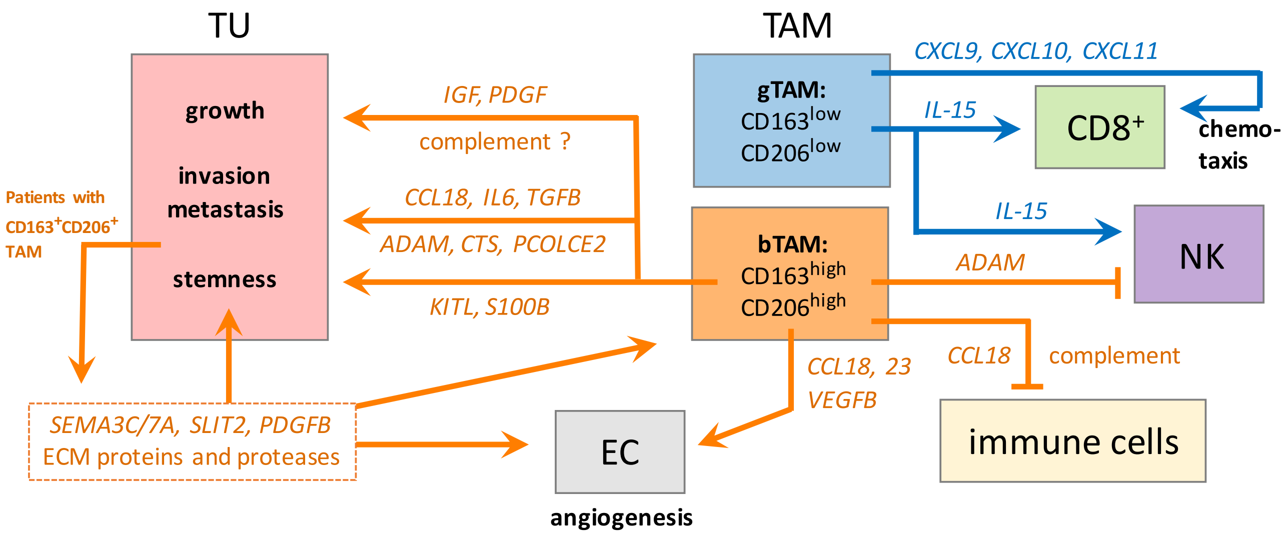

Supplementary Figure 11.

Scheme depicting the contrasting roles of distinct TAM subsets in HGSOC biology. TAM-secreted mediators are grouped according to the data in Fig. 6: blue, gTAM, i.e., synthesis correlated with low *CD163* and *MRC1/CD206* expression (favorable clinical outcome); red, bTAM, i.e., synthesis correlated with high *CD163* and *MRC1/CD206* expression (poor clinical outcome). The scheme includes only proteins selectively expressed by gTAM or bTAM. Proteins predicted from RNA-Seq data are italicized. $CD8^+$: cytotoxic T cell; EC, endothelial cell; NK: natural killer cell.
